# Evolving Role of Conjugated Polymers in Nanoelectronics and Photonics

**DOI:** 10.1007/s40820-025-01748-7

**Published:** 2025-04-24

**Authors:** Amaan Chougle, Ayman Rezk, Syed Usama Bin Afzal, Abdul Khayum Mohammed, Dinesh Shetty, Ammar Nayfeh

**Affiliations:** 1 Department of Electrical Engineering, Khalifa University, 127788, Abu Dhabi, UAE; 2 Department of Chemistry, Khalifa University, 127788, Abu Dhabi, UAE; 3 Center for Catalysis and Separation (CeCaS), Khalifa University, 127788, Abu Dhabi, UAE; 4 Research and Innovation Center for Graphene and 2D Materials (RIC-2D), Khalifa University, Abu Dhabi, UAE

**Keywords:** Conjugated polymers, Molecular engineering, Organic photonics, Organic electronics

## Abstract

Conjugated polymers (CPs) have emerged as an interesting class of materials in modern electronics and photonics, characterized by their unique delocalized π-electron systems that confer high flexibility, tunable electronic properties, and solution processability. These organic polymers present a compelling alternative to traditional inorganic semiconductors, offering the potential for a new generation of optoelectronic devices. This review explores the evolving role of CPs, exploring the molecular design strategies and innovative approaches that enhance their optoelectronic properties. We highlight notable progress toward developing faster, more efficient, and environmentally friendly devices by analyzing recent advancements in CP-based devices, including organic photovoltaics, field-effect transistors, and nonvolatile memories. The integration of CPs in flexible sustainable technologies underscores their potential to revolutionize future electronic and photonic systems. As ongoing research pushes the frontiers of molecular engineering and device architecture, CPs are poised to play an essential role in shaping next-generation technologies that prioritize performance, sustainability, and adaptability.

## Highlights

- This review offers an overview of recent advancements in conjugated polymers (CPs), with a thorough discussion of their molecular engineering. Key electronic properties are put forth that complement traditional inorganic semiconductor devices.
- Key concepts and innovations in molecular engineering are discussed, highlighting advancements that improve device performance, with a particular focus on photovoltaics, organic field-effect transistors, and nonvolatile memory devices.
- The current challenges in fabricating CP-based devices are explored, along with anticipated future developments and growing market demand.

## Graphical abstract

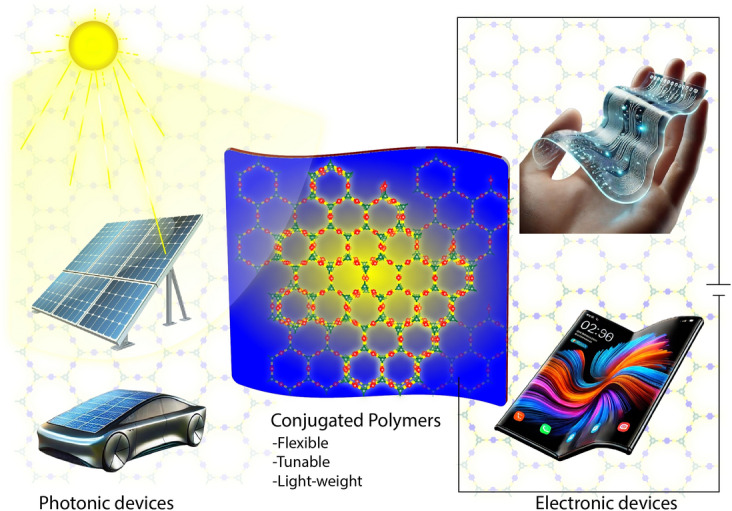

## Introduction

Conjugated polymers (CPs) have significantly impacted the field of electronic and photonic devices in the twenty-first century, offering a versatile class of materials with properties that complement traditional inorganic semiconductors [1]. Traditional materials including silicon (Si), germanium (Ge), and gallium arsenide (GaAs), which have dominated research and industry and are known for their high performance and stability in devices like computer processors and solar panels, often face challenges including rigidity, high production costs, environmental concerns, and performance instability under extreme conditions. CPs’ mechanical flexibility and tunable bandgaps, with their easy and low-cost processing [2], have opened new avenues for device fabrication and application, including organic light-emitting diodes (OLEDs), organic photovoltaics (OPVs) [2–4], organic field-effect transistors (OFETs) [5, 6], and bioelectronics [7–11], making them especially useful for flexible and wearable devices [12]. However, the path to widespread adoption of CPs has challenges [13]. They often exhibit lower stability and efficiency than traditional semiconductor devices, facing chemical and thermal stability limitations, power conversion efficiency, long-term operational stability, and environmental resilience [14]. CPs are susceptible to degradation under environmental factors such as heat, humidity, and UV exposure, which impacts the lifespan and reliability of CP-based devices. Furthermore, achieving consistent film quality and integration with existing CMOS-based technologies present processing challenges. To address these issues and reduce costs associated with fabrication, advanced fabrication techniques, like molecular engineering and nanostructuring, alongside hybrid approaches combining CPs with nanomaterials and inorganic semiconductors [15] are being explored. Innovations in solution-based and roll-to-roll printing [16] are also under development to enhance stability, improve charge transport properties [17, 18], and achieve uniform, high-quality films [19].

The ability to design and synthesize CPs with tailored material properties is pivotal to their application across various technologies. However, achieving a balance between solubility, mechanical stability, and charge transport has proven challenging, often resulting in tradeoffs discussed in multiple reviews [20–22]. Researchers have developed innovative design methodologies centered around molecular engineering, manipulating the polymer backbones and side chains to fine-tune electronic properties for specific properties. One approach includes altering the polymer backbone to enhance stability while introducing ferroelectric properties for OFETs [23]. Another method includes incorporating a redox-active site in the backbone to enhance proton storage capability while maintaining high crystallinity [24]. Additionally, increasing the side-chain alkyl groups has optimized thermomechanical properties while attaining isotropic charge mobilities under strain [25, 26]. To further refine the CP performance predictions, Alesadi et al. (2022) developed a machine learning-integrated molecular dynamics model capable of predicting thermomechanical properties and local charge mobilities based on molecular features such as side-chain fractions, isolated rings, fused rings, and bridged rings [27].

Optoelectronics play a vital role in advancing technologies used in energy conversion, sensing, and communication, and CPs have made substantial contributions by enabling advancements in OPVs [28–30], OLEDs [31], organic photodetectors [32], and laser power converters [33]. The materials employed in these applications must combine attributes of mechanical flexibility, suitable electronic properties, high efficiency, low toxicity, and affordability [34, 35]. Due to the presence of delocalized π-electron systems, they can modulate their optical bandgaps through molecular modification, thereby adjusting their photon absorption and emission characteristics [36]. This is essential for applications like OLEDs, which require specific emission wavelengths, or photodetectors and solar cells, which benefit from broad-spectrum absorption. One-dimensional (1D) CPs possess anisotropic electrical properties and are amenable to solution-based processing, whereas two-dimensional (2D) CPs allow for extended π-conjugation in planar structures, enhancing electronic conductivity and charge carrier mobility [37–39]. Moreover, molecular engineering plays a key role in their enhancement, and recent approaches include incorporating additional electron-donating [40] or electron-withdrawing [41, 42] substituents in donor–acceptor moieties, which have resulted in OPVs with efficiencies of 19% acquiring high crystallinity, green-solvent processability, and blade-coating capability paving the way for upscaling and commercialization. Additionally, increasing conjugation length in the polymer backbone helps in tuning the highest occupied molecular orbital (HOMO) and lowest unoccupied molecular orbital (LUMO) levels to achieve the desired optical properties [43].

In bioelectronics, CPs have facilitated remarkable innovations, leading to the development of wearable and implantable devices for real-time monitoring and therapeutic applications. Polymers like poly(3-hexylthiophene) (P3HT), poly(3,4-ethylenedioxythiophene): polystyrene sulfonate (PEDOT:PSS), polyaniline (PANI) and polypyrrole (PPy) have been extensively studied for biointerfacing due to their biocompatibility, electrical conductivity, and mechanical properties that complement biological tissues [7, 8]. Furthermore, CPs have demonstrated potential in bioimaging, cancer/gene therapy, and drug delivery attributed to their versatile $\pi$-conjugation systems, amphipathic characteristics, cationic functionalities, and the capacity to generate reactive oxygen species (ROS) upon photoexcitation upon light exposure [9].

As the field of CPs rapidly evolves, these materials are positioned to profoundly influence electronic and photonic technology landscapes. From enhancing the efficiency of photovoltaic systems and FETs to enabling breakthroughs in biomedical engineering, CPs are foundational to future technological advancements. Their customizable properties and facilitation of eco-friendly fabrication processes position them as viable alternatives to inorganic materials in next-generation electronics. Several comprehensive reviews have been conducted on CPs [44, 45]. This review, however, offers an update on the latest developments in CPs designed for various electronic and photonic applications. The following sections categorize different types of CPs and discuss the novel design strategies employed in their development. Subsequently, we provide an in-depth analysis of their technological applications, highlighting performance parameters and potential impacts.

## Conjugated Polymers

### One-Dimensional (1D) and Two-Dimensional (2D) CPs

**Fig. 1:**
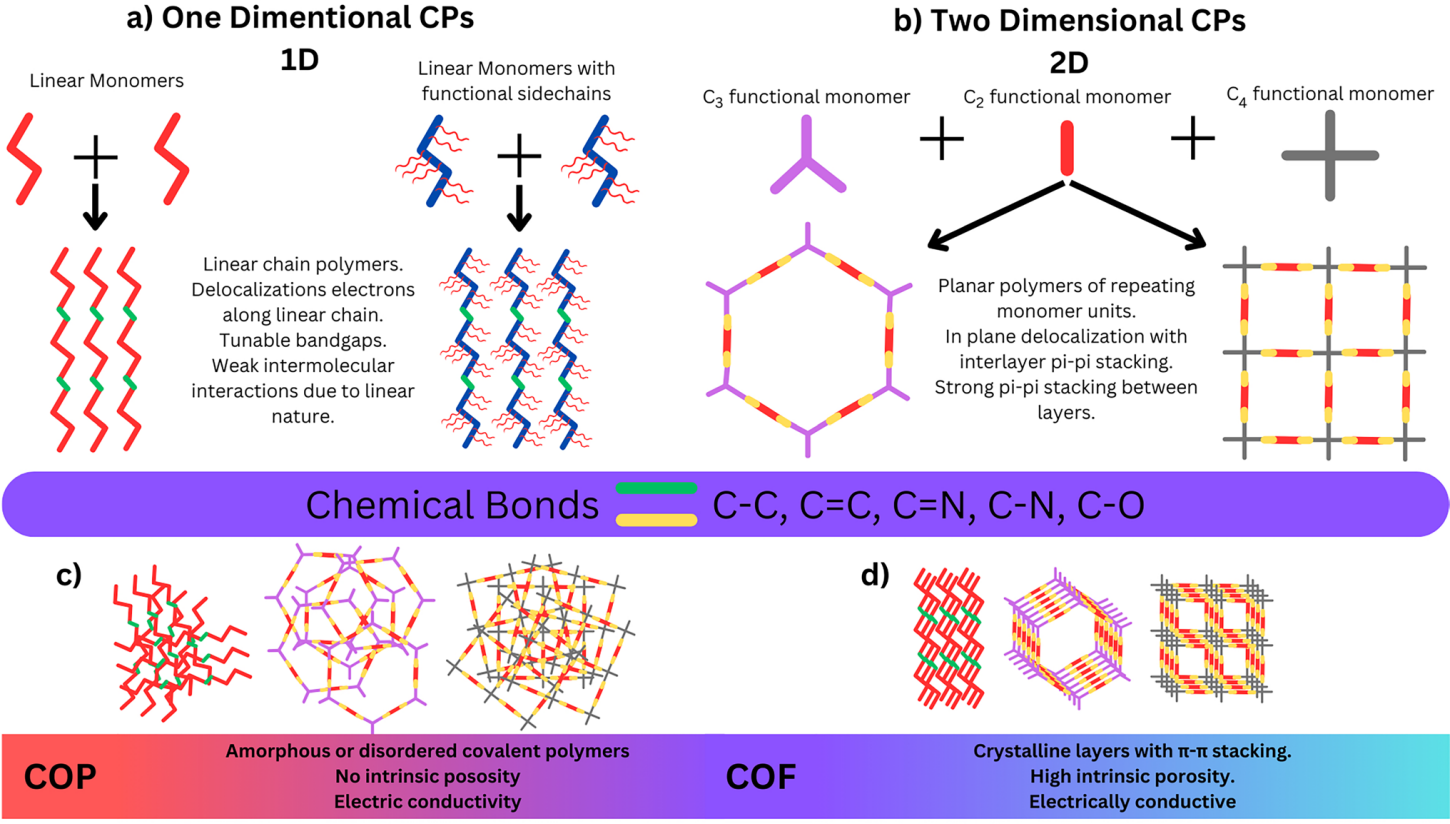
**a** Diagrammatic representation of the formation 1D polymers from linear monomers and 1D polymers with side-chain functionalizations; **b** Diagrammatic representation of the formation of 2D polymers from symmetric monomers (C_3_, C_2_, and C_4_); **c** Graphical representation of COPs shows random ordered frameworks and **d** graphical representation of COFs shows the ordered frameworks with intrinsic pores

CPs have been developed over many years and exhibit mainly 1D and 2D structures as illustrated in Fig. 1. The various dimensional architectures of the CPs depend on the type of symmetry, geometry, and functional groups of monomers. Importantly, 1D CPs are geometrically linear in shape as illustrated in Fig. 1a. In most cases in 1D CPs, monomers have two functional groups that propagate the reaction in a 1D direction and form chain-like polymers, whereas 2D polymers are planar and layered materials composed of repeating organic units in a 2D plane Fig. 1b. Generally, 1D and 2D polymers can exist in either crystalline or amorphous states, depending on their building blocks’ symmetry, the nature of their functional groups, and the reaction conditions. Polymers with a regular arrangement of covalently connected molecules display intrinsically ordered pores and the precise integration of functional moieties. These ordered frameworks are known as covalent organic frameworks (COFs) (Fig. 1d). Additionally, the COFs possess weak intermolecular interactions (π-π stacking) between the 2D organic layers, creating the material’s intrinsic structural features. Meanwhile, 1D and 2D polymers without a distinct ordered molecular structure are called covalent organic polymers (COPs) (Fig. 1c). COFs and COPs have been widely applied for optoelectronic applications due to their intrinsic structural features.

Interestingly, 1D CPs possess anisotropic optical, conductive, and mechanical properties, tunable bandgaps, and solution processability, bringing them into the spotlight for organic electronics, optoelectronics, and thermoelectric applications [46, 47]. In these systems, the delocalized electrons are forced to traverse predominantly along the linear chain of atoms, and the movement in any other direction is restricted due to geometrical constraints. The chain lengths are relatively small, allowing the formation of delocalized energy states in one direction. Various factors resulting from the chemical nature of the structure affect the movement of electrons within the linear chain, generating only partially delocalized energy states for the valence electrons, which impart semiconducting properties [48].

Meanwhile, 2D CPs, especially in COFs, allow for the planar delocalization of electrons. Due to this in-plane conjugation and interlayer π-π stacking capability, 2D CPs attain unique band structures and allow for thin-film formation, which makes them desirable for organic FETs, memristors, chemiresistors, and photodetectors. Meanwhile, 1D CPs have an interchain hopping defect for valence charge carriers which retards the conductivity. This is resolved within 2D CPs, enhancing the conductivity and mobility of charge carriers in the active layers [49]. Depending on the stacking level produced by reaction conditions, 2D CPs are formed by periodically connected monomers through covalent bonding and can exist as a few or multilayers. The advantages of 2D CPs include layers having atomic thickness, large specific surface area, and mechanical flexibility, which are attained from the planar structure of the polymer.

### Design and Engineering Strategies for CPs

The optoelectronic properties of CPs depend on several factors, including chemical linkages, the presence of various functional groups, and the extent of conjugation. Typically, CPs are synthesized from monomers through covalent polymerization as illustrated in Fig. 1. The strategic selection of suitable monomers is crucial in fabricating CPs with desired properties. Next, the nature of chemical linkages and reticular design plays a critical role in determining the optoelectronic behavior of CPs. The chemical linkages particularly influence the extent of conjugation in 1D or 2D CPs. If a chemical linkage disrupts conjugation between monomers, the resulting polymers exhibit limited conjugation and subsequent properties. Thus, controlling conjugation relies heavily on the induced chemical bonds. For example, olefin-linked (C = C) polymers exhibit better electronic conductivity compared to imine (C = N) bonded polymers [50]. In the latter case, electron density is more polarized toward nitrogen due to its electronegativity, leading to less efficient electron transfer. In contrast, the olefin bond lacks such polarization, allowing for smooth electron transfer.

Additionally, integrating functional groups into polymers can significantly modify their physicochemical properties, which include tuning their bandgaps. In addition, multiple design strategies have been developed and tested to acquire specific properties of the polymer that could make it suitable for electronic and photonic applications. These properties include the porosity, solubility and processability, crystallinity, and flexibility of the polymer [51–54]. Notably, controlling these factors is essential since they impact the charge/carrier transport in the material, the ease of synthesis and manufacturability with common solvents, scalability, and physicochemical stability of the CPs. Researchers have invented significant novelties in altering and enhancing these properties in the past few years, leading to efficient and economical thin films and devices. The basic rule behind attaining variable properties for polymers lies in engineering the polymer’s backbone and side chains.

The properties of CPs can be tuned by the engineering of the backbone of the polymers through the integration of suitable functional groups. Backbone engineering involves selecting specific C_2_, C_3_, and C_4_ monomers (Fig. 1b), altering the conjugation length, and introducing heteroatoms into the $\pi$-conjugated system to attain device-favorable properties. Once the monomers are finalized, other important properties, such as solubility, processability, stability, and interaction with other materials, are molded by the side chains of the backbone structure [4, 55, 56]. Additional pendant groups on the side chain, such as the ones shown in Fig. 1 with the 1D CP, have been shown to enhance the absorption characteristics and induce bandgap modulation capability.

Incorporating bulky pendant groups into the backbone has been shown to enhance the solubility and processability of CPs. This improvement is due to weak molecular interactions between the solvent molecules and the pendant groups. Moreover, in COFs, introducing bulky pendant groups creates steric hindrance between the layers, reducing interlayer stacking and resulting in a few-layer COF structure that promotes solubility [57]. This strategy exhibits enhanced electron mobility of 3200 cm^2^ V^−1^ s^−1^, comparable to graphene. The selection of monomers also leads to the attainment of high conductivity, as demonstrated by Wang et al. (2020) [58]. Here, the authors design a polymer of phthalocyanine and anthraquinone derivates, which attain an abundance of electrons and delocalization facilitated by the fluorinated phthalocyanines, while anthraquinones facilitate charge transfer.

Furthermore, the field of backbone engineering can be broken down into two categories based on developing outcomes. These are organic ionic-electronic conductors (OIEC) and donor–acceptor alternating conjugated polymers (DAACPs). The OIECs are materials that acquire dual capability of ionic and electronic conduction, designed by a mixture of conjugated and insulating polymers [7, 59, 60]. A significant OIEC is a mixture of PEDOT with PSS, which not only induces dual conduction, but the presence of PSS also enhances the solubility of PEDOT, which initially was not soluble once polymerized [7]. On the other hand, DAACPs are a mixture of donor and acceptor polymers that acquire a tunable optical bandgap by changing the acceptors and are highly applicable in photovoltaic applications [13, 61].

In addition to the backbone, their side chains also play a crucial role in managing the electrical and optical properties. The side chains are bulky alkyl groups of various lengths that act as linkers and exist at the ends of the D-A units. These can enhance the bonding between multiple D-A blocks and solvent molecules. The introduction of side chains allows for the bending and twisting of backbone structures, which have been shown to enhance the stacking of layers, providing high stability and crystallinity in addition to increasing the fluorescence, charge transport, and UV–Vis’s absorption of the polymers [54, 62]. The strategic design of CPs, incorporating donor–acceptor architectures, side-chain engineering, and backbone modifications, offers a versatile approach to tailoring their optoelectronic properties. Properties such as bandgap, charge carrier mobility, and absorption spectra can be finely tuned by carefully selecting molecular structures and optimizing conjugation lengths. These design strategies are anticipated to yield CPs with enhanced performance in organic photovoltaics, sensors, and other advanced electronic applications, paving the way for next-generation organic semiconductor technologies.

## CP-Based Devices

### Photovoltaics

CPs have emerged as a versatile class of materials with promising applications in various photonic technologies such as photovoltaics, photodetectors, and laser power converters [56, 63, 64]. In this section, we will focus specifically on their photovoltaic applications and how CPs have led to significant advancements in energy conversion. We aim to explore the recent developments and key findings related to using CPs to enhance the efficiency and performance of solar cells, as well as potential future directions in this research field. Additionally, CPs offer an advantage over perovskite materials, being less hazardous than lead-based perovskites and exhibiting better long-term stability against humidity and temperature [65–69].

Numerous combinations of CPs have been designed and synthesized for all-polymer solar cells. As mentioned earlier in the design section, for donor–acceptor bulk-heterojunctions (D-A BHJs) in DAACPs, diimide-based polymers have shown promising results along with multiple other D-A combinations, which have been demonstrated over the last few years, which are summarized in Table 1. The performance parameters for the specific D-A-based solar cells are highlighted, which include open-circuit voltage (*V*_oc_), short circuit current density (*J*_sc_), fill factor (FF), and power conversion efficiency (PCE). Additionally, significant progress has been made in single-component organic solar cells (SCOSCs), which will be discussed in the following section.

**Table 1:** Performance metrics of state-of-the-art DACCP-based solar cells

Active Layer	*V*_OC_ (V)	*J*_SC_ (mA cm^−2^)	*FF* (%)	*PCE* (%)	Ref/Year
PT8:F10	1.04	12.58	69	9.04	[77]/2021
PM6:PNDI-2 FT-TR10	0.92	17.32	67	10.71	[70]/2021
PTz-BI-Si:P(NDIHDDT-T2)	0.86	16.4	78.1	11.2	[71]/2022
PBDB-T-2 F:N3:P(NDI2OD-T2)	0.86	24.3	67.1	14.04	[72]/2022
PTB-7-Th:PBTI3-T	1.03	14.88	58.46	8.98	[43]/2022
P3HT:DCNBT-IDT	0.65	16.7	68	7.35	[76]/2022
PBQx-TF:eC9-2 Cl	0.877	27.1	80.2	19.1	[74]/2024
PM6:L8-BO with 1% B6 Cl	0.895	27.4	80.6	19.8	[75]/2024
D18:L8-BO with 0.5% B6 Cl	0.919	27.2	80.7	20.2	[75]/2024
PM1:PTQ10:m-BTP-phC6	0.876	27.57	80.8	19.51	[78]/2024

#### Donor–Acceptor Alternating Conjugated Polymers in Photovoltaics

**Fig. 2:**
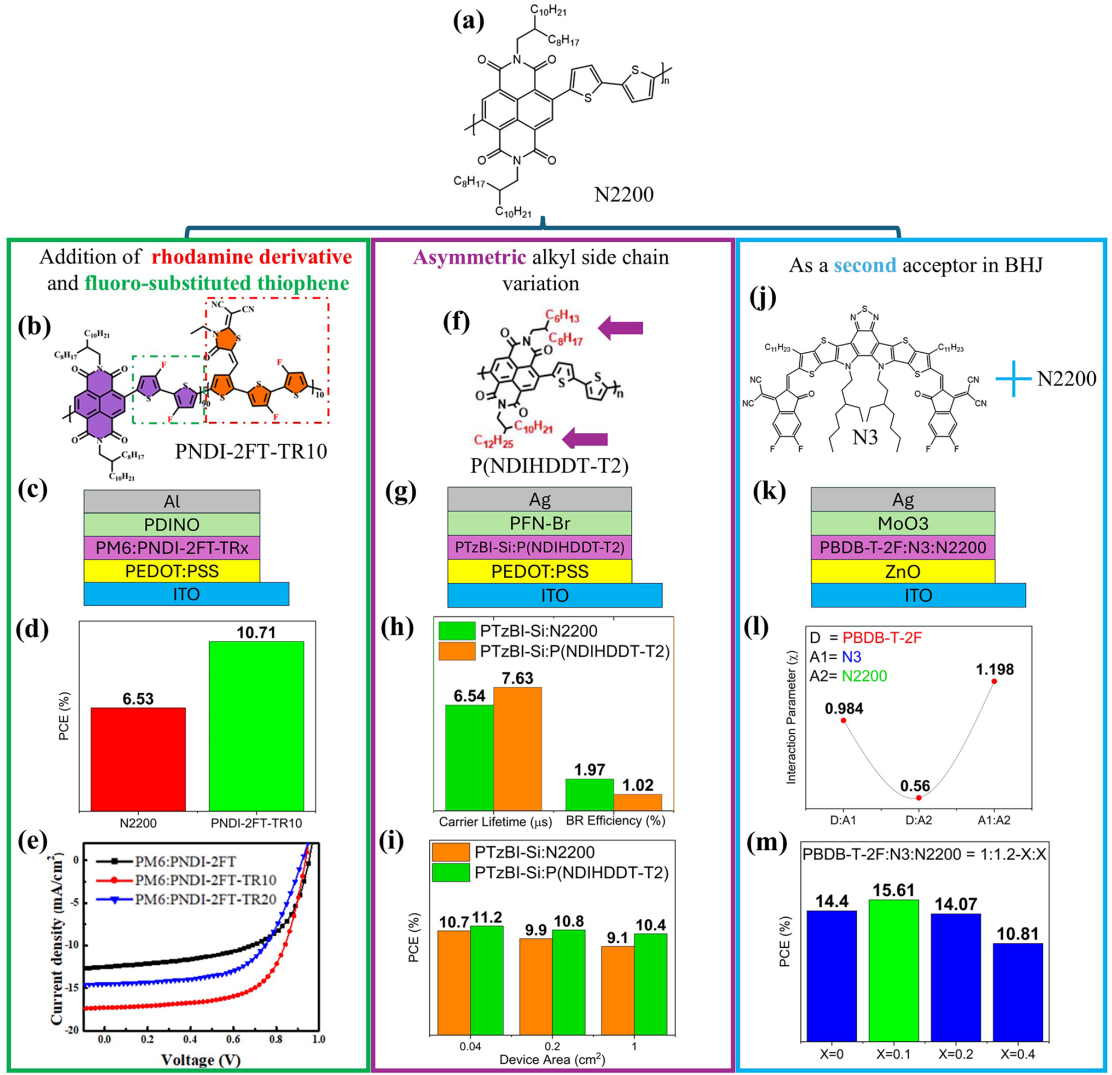
**a** Chemical structure of N2200. **b–e** Chemical structure of modified N2200 polymer, device architecture, PCE comparison between N2200 and modified polymer-based devices, and *J-V* curves of devices with various blends (**b** and **e** Reproduced with permission from Ref. [70] Copyright 2021 American Chemical Society). **f–i** Chemical structure of asymmetric N2200 polymer, device architecture, comparison of carrier lifetime and BR efficiency of devices, and the PCE comparison for various device sizes (**f** Reproduced with permission from Ref. [71] Copyright 2022 Elsevier). **j–m** Chemical structures of 2 acceptors in the BHJ, device architecture, Flory–Huggins interaction parameters of three blends in the BHJ, and PCE comparison for various BHJ blend ratios (**j** Reproduced with permission from Ref. [72] Copyright 2022 Elsevier)

In recent research, naphthalene diimide (NDI) has been incorporated as an effective acceptor unit in various BHJs [70–72]. Typically, NDI-derived acceptors have low absorption coefficients, resulting in PCEs of no more than 10, even when paired with highly efficient donors [73]. The primary issue is the low thermodynamic miscibility between the donor and acceptor, which causes increased phase separation and inefficient charge separation. Multiple research papers deal with resolving this issue, and a lot is based on the NDI acceptor, P(NDI2OD-T2) (poly[[N,N’-bis(2-octyldodecyl)-naphthalene-1,4,5,8-bis(dicarboximide)−2,6-diyl]-alt-5,5’-(2,2’-bithiophene)]), more commonly known as N2200 (Fig. 2a). Chen et al. (2021) [70] synthesized two NDI acceptors (PNDI-2 FT-TR10 and PNDI-2 FT-TR20), which show enhanced miscibility with the PM6 donor. The authors achieved this by designing terpolymers through random polymerization of N2200. This involves the strategic addition of 2-(1,1’-dicyanomethylene)−4-(3-thienylmethylene) rhodamine (TR) into the backbone, which allows the reduction of aggregation of the copolymer and induces stronger absorption characteristics. The substitution of bithiophene (2 T) blocks in N2200 with 3,3’-difluoro-2,2’-bithiophene (2 FT) groups adds interchain and intrachain noncovalent interactions that boost the charge transport characteristics. These variations are illustrated in Fig. 2b. This results in a higher PCE than the N2200-based device and shows better *J-V* characteristics as illustrated in Fig. 2d, e. The addition of 10 TR (PNDI-2 FT-TR10) was found to be optimum, as a further addition to 20 (PNDI-2 FT-TR20) increased the bimolecular recombination resulting in lower performance as contrasted in Fig. 2e [70].

Another strategy explored by Li et al. (2022) [71] is the synthesis of a side-chain variation of N2200, P(NDIHDDT-T2), which incorporates an asymmetric side chain. The authors aimed to replace the two 2-octyldodecyl (OD) groups with less bulky 2-hexyldodecyl (HD) and a 2-decyltetradecyl (DT) groups (Fig. 2f). The chemistry behind this approach is that shorter side chains increase aggregation in the polymer chain, enhance crystallinity, and alleviate layering and the π-π stacking distance, promoting charge transport [71]. Moreover, since reducing side-chain lengths can negatively impact the solubility, only one of the side chains is reduced in length while the other is increased to achieve a balance between crystallinity and solubility as highlighted in Fig. 2f. This new acceptor, when blended with PTz-BI-Si donor, shows enhanced performance compared to N2200-based all-PSCs of various sizes as illustrated in Fig. 2i. Moreover, this strategy enhances the carrier lifetime in the device by suppressing the bimolecular recombination (BR) efficiency that is 1 inhibited by the short HD side chain (Fig. 2h) [71].

Besides its usage as a standalone acceptor, incorporating N2200 as a second acceptor shows enhanced photovoltaic performance [72]. Herein, the second acceptor enhances the PBDB-T-2 F:N3 BHJ system by forming almost miscible blends with the donor. Figure 2l depicts the Flory–Huggins interaction parameters ($\upchi$) for the various blends in the BHJ. A smaller value of $\chi$ indicates critical stability, and higher values indicate increased phase separation and thermodynamic instability. The PBDB-T-2 F:N2200 blend shows better miscibility and creates intricate channels within donor crystallites. These channels facilitate efficient charge dissociation, reduce recombination, and provide electron transport pathways. However, increasing the N2200 ratio beyond an optimum (X = 0.1) increases the charge carrier recombination and reduces the PCE, as illustrated in Fig. 2m for various BHJ blend ratios [72].

NDI also has excellent electron transport properties and high transmittance in the visible region, making it promising for use as a transport layer. Yu et al. (2024) phenyl-substituted NDI-based cathode interlayer (CIL), which exhibited exceptional properties due to its structural modifications [74]. Firstly, the phenyl substitutions on the 2,6-positions of the NDI structure led to a reduced packing distance of 3.44 Å between the polymer layers. A high intrinsic carrier density and a favorable work function also facilitate efficient charge extraction and transport. The material’s self-doping characteristics also benefit it from attaining a sustainable PCE even at thicker films of 100 nm. When incorporated into a PBQx-TF:eC9-2 Cl BHJ, the resulting device achieved a PCE of 19.1%, outperforming other previously reported CILs such as PDINN and PNDIT-F3 N [74].

**Fig. 3:**
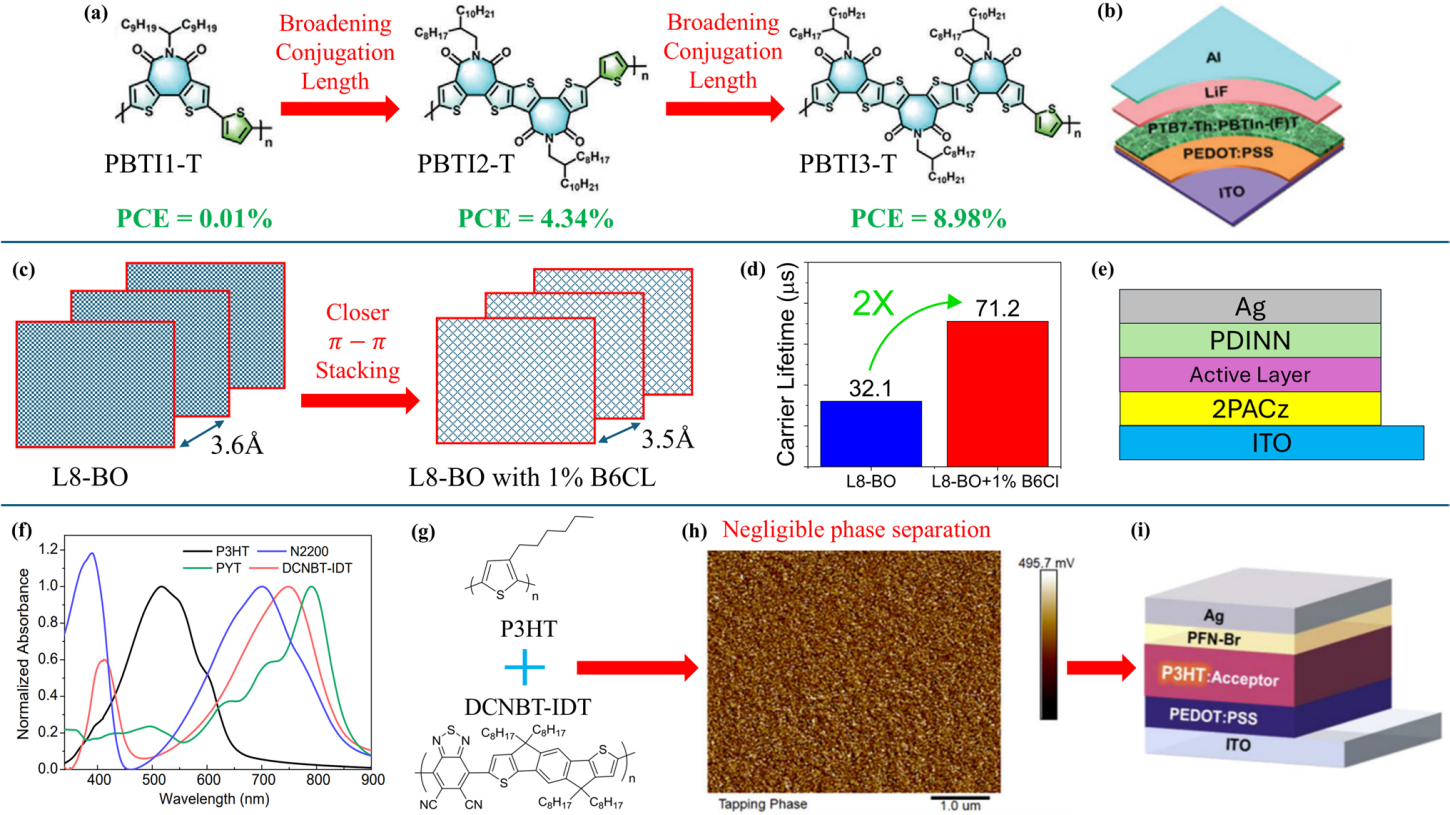
**a–b** Chemical structures of polymers with their reported PCEs in the illustrated device architecture (**a** and **b** Reproduced with permission from Ref. [43] Copyright 2022 John Wiley and Sons). **c–e** Illustration showing the closer packing of planar acceptor layers and the enhancement of carrier lifetime with the used device structure [75]. **f–i** Absorption spectrum of the donor and acceptors, chemical structures of the primary acceptor and donor, phase images of the blend film, and the device architecture used (**f-i** Reproduced with permission from Ref. [76] Copyright 2022 John Wiley and Sons)

In addition to the NDI-based polymers, researchers have synthesized many novel donors and acceptors that, despite limited use in broader literature, have still impacted photovoltaic research. These CPs feature distinctive design strategies in their structures that make them stand out. Liu et al. (2022), in their work [43], reported an acceptor PBTI3-T that is based on the ladder-type heteroarene BTI (bithiophene imide). The distinctive design here involves the introduction of additional thiophene and 3,4-fluorothiophene units into the backbone, which results in broadening of conjugation length (Fig. 3a) and tuning the HOMO and LUMO levels. The structure of these polymers, characterized by fused aromatic rings and thiophene units, contributed to their extended conjugation lengths, further improving their electronic characteristics of exciton dissociation and charge transport. When blended with PTB-7-Th donor, the polymers showcase a direct proportionality between PCE and conjugation length as illustrated in Fig. 3a, pointing out the importance of extended conjugation in polymers for photovoltaic applications. In addition, the bulky alkyl side chains induce curvature into the backbone which enhances the solubility and blend morphology of the polymers [43].

As depicted in Table 1, recent advancements in all-polymer solar cells have achieved significant progress, reaching 20% PCEs. Our observation suggests that there has been little architectural innovation and that research primarily focused on materials over the past few years.

#### Single Component Organic Solar Cells

**Fig. 4:**
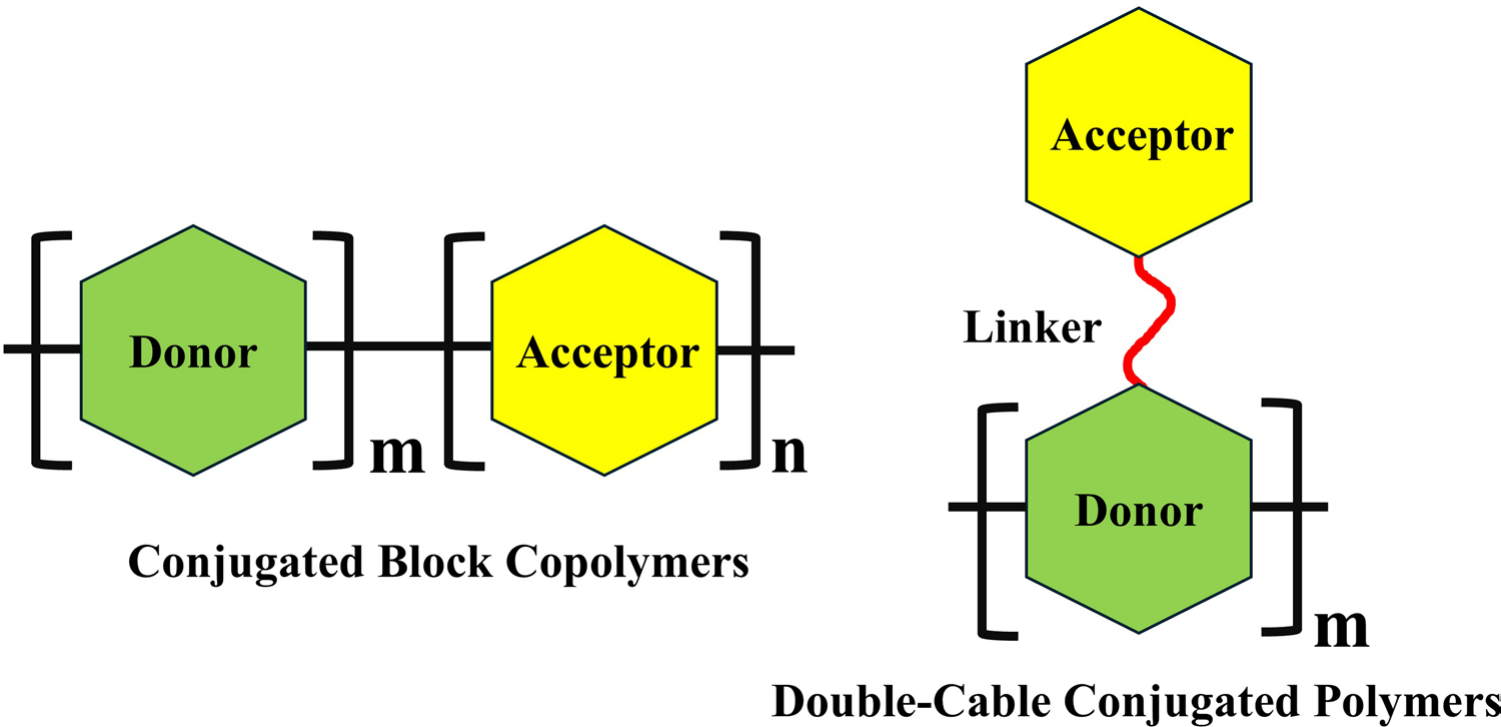
Illustrations of chemical structures for CBCs and DCCPs

DAACPs consist of physical mixtures of donor and acceptor components. While they perform well, they have inherent limitations. The physical mix can lead to significant phase separation in the bulk heterojunction, reducing overall performance. Over time, exposure to heat and light can increase these defects and potentially shorten the charge carrier lifetime. To address these issues, a key research milestone is the chemical bonding of donor and acceptor moieties within a single molecule to enhance device stability and carrier lifetime. SCOSCs achieve this through conjugated block copolymers (CBCs) or double-cable conjugated polymers (DCCPs), which mitigate phase separation causing lifetime and stability issues [79–82]. CBCs and DCCPs are distinctive in their structures and charge transport properties, as illustrated in Fig. 4. CBCs feature alternating donor and acceptor units covalently bonded within a single molecule. The charge is transported via the corresponding donor or acceptor units. DCCPs have a single backbone, usually of donor characteristics, with pendant or sidechain acceptor units attached to the backbone. Here, the electrons are transported through the backbone while the holes are transported through the sidechains.

**Fig. 5:**
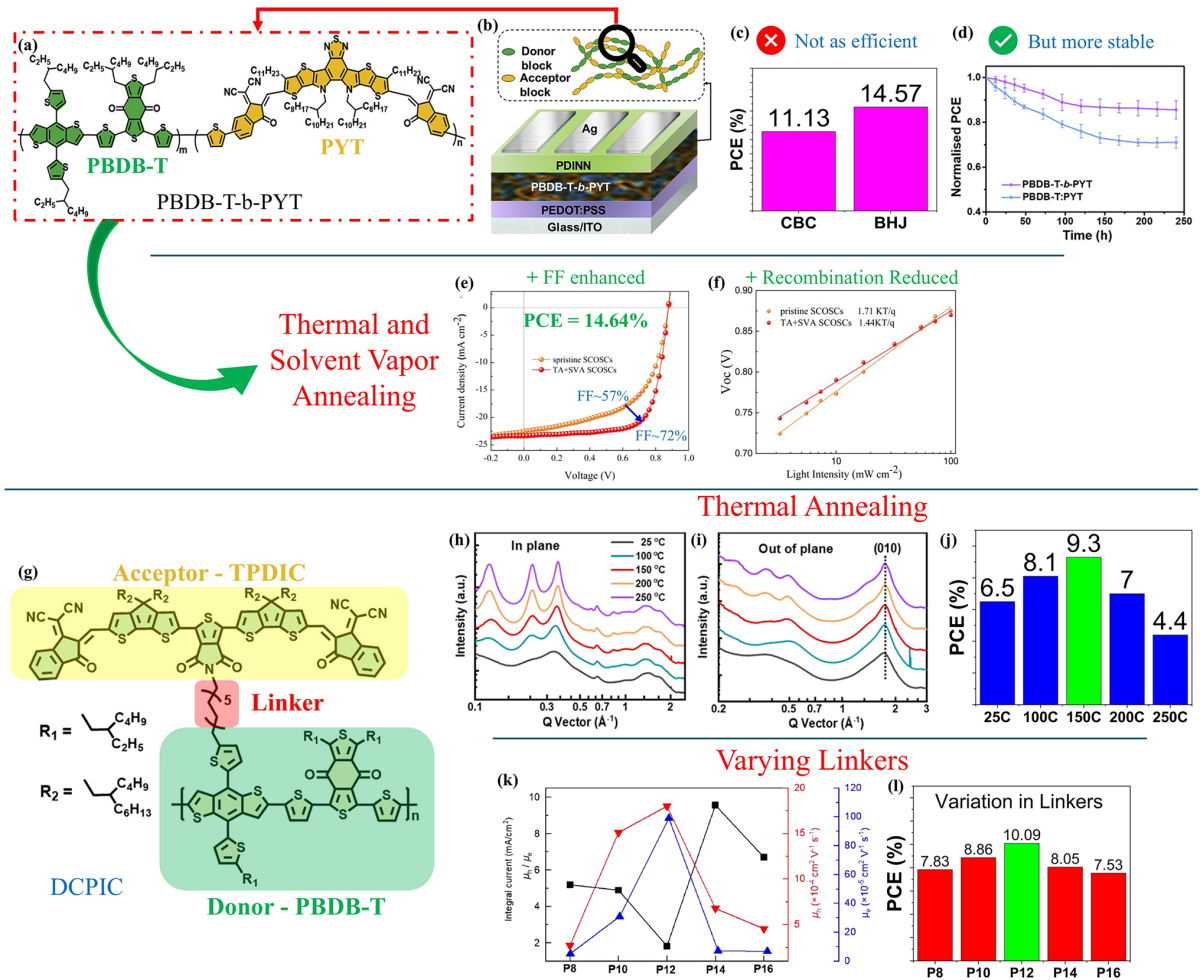
**a-d** Chemical structure of CBC PBDB-T-b-PYT, device architecture used in the study, PCE comparison between CBC and BHJ for the polymer, and normalized PCE vs time with light-soaking illustrating the photostability of the device (**a, b** and **d** Reproduced with permission from Ref. [83] Copyright 2021 Elsevier). **e–f** *J-V* curve and *V*_oc_ dependence on light intensity plot for pristine and optimized SCOSC based on PBDB-T-b-PYT CBC (**e** and **f** Reproduced with permission from Ref. [84] Copyright 2024 American Chemical Society). **g** Chemical structure of DCCP DCPIC [85]. **h–j** The GIWAXS profiles along the in-plane and out-of-plane direction, and the PCE of devices as a function of the annealing temperature (**g–i** Reproduced with permission from Ref. [85] Copyright 2024 Elsevier). **k–l** Hole and electron mobilities and PCE of DCCP-based devices with various linkers (**k** Reproduced with permission from Ref. [86] Copyright 2024 Elsevier)

SCOSCs have certain disadvantages and advantages over DACCPs. For instance, the work by Wu et al. (2021) highlights how CBC-based SCOSC devices are less efficient than their BHJ counterparts, however, they attain higher stability over a longer period [83]. The authors synthesize the CBC PBDB-T-b-PYT (Fig. 5a), which is a combination of the donor PBDB-T and acceptor PYT, by a one-pot synthesis technique. From Fig. 5b, we can observe a reduction in the performance of the SCOSC which is attributed to the reduced carrier lifetime from intramolecular charge transfer. This hypothesis is put forward by observing a higher reduction in PL intensity in the CBC as compared to the BHJ. However, the CBC device demonstrated improved light stability due to reduced photoinduced degradation. The better stability arises from the CBC combining donor–acceptor into a single molecule, resulting in lower morphological degradation over time when soaked in light. The BHJ, being a physical mixture, shows higher molecular ordering and aggregation, degrading the PCE at a higher rate over time.

This drawback has recently been overcome by Liu et al. (2024) wherein they optimize the SCOSC by thermal annealing and solvent vapor annealing [84]. This strategy aims to reduce the trap-assisted recombination and improve charge transport characteristics. As a result, we can observe that the optimized device has a PCE comparable to the BHJ due to enhanced FF (Fig. 5e), while also maintaining the long-term stability of the SCOSC. The thermal and solvent annealing processes yield thin films with better morphologies and reduced recombination as observed from the Voc vs light intensity plot slope in Fig. 5f (slope closer to 1 signifies lower trap-assisted recombination). Moreover, the stability analyses in this study revealed an 80% retainment of initial PCE with the optimized device, as compared to 60%–70% for the pristine SCOSC and BHJ-based devices. Another innovative CBC optimization strategy is adding a second acceptor into the copolymer. Wu et al. (2022) demonstrated that the covalently bonded donor and acceptor units in the CBC are restricted in segmental motions, leading to free volumes within the structure that hinder crystallinity and reduce performance [87]. Herein, the authors introduce a second acceptor, Y6, into the CBC PM6-b-PYIT, which serves as an additive to improve morphology. Y6, which exhibits structural similarity to the acceptor PYIT and has superior miscibility with it as indicated by the Flory–Huggins interaction parameter, is used to fill the voids and reorganize the acceptor portion for compact molecular packing and suppression of segmental motions during aging to enhance stability. This strategy exhibits an amazing SCOSC performance and has the highest PCE reported with CBCs (Table 2).

**Table 2:** Summary of performance metrics for SCOSCs

Active Layer	*V*_OC_ (V)	*J*_SC_ (mA cm^−2^)	*FF* (%)	*PCE* (%)	Ref/Year
PBDB-T-b-PYT	0.914	19.25	63.3	11.13	[83]/2021
PBDB-T-b-PTY6-γ	0.9	19.07	59	10.51	[81]/2022
PM6-b-PYIT:Y6	0.93	23.46	71.5	15.55	[87]/2022
as-DCPIC	0.77	21.23	62	10.09	[82]/2022
PB-b-PYCl-2	0.87	22.33	69.3	13.42	[88]/2023
PBC12-T	0.93	9.78	61	5.56	[89]/2023
SC-1	0.855	18.05	60.5	9.35	[80]/2024
P12	0.767	21.2	62.3	10.09	[86]/2024
DCPIC	0.806	18.97	60.8	9.3	[85]/2024
PBDB-T-b-PYT	0.877	23.35	71.4	14.64	[84]/2024

Like in CBCs, the thermal annealing strategy has also been proven to apply to DCCPs. Zhang et al. (2024) have explored the relationship between the crystallinity of the active layers and the performance of the SCOSCs [85]. They exposed the active layers to thermal annealing at various temperatures, with higher temperatures leading to higher crystallinity than the lower temperatures. Within the temperature range of 25–250 °C, 150 °C exhibited the optimum device performance due to enhanced phase morphology with an RMS roughness of only 0.58 nm, which is the smallest in this study. In addition, GIWAXS measurements were conducted, and the results revealed an increasing trend in lamellar peaks in the in-plane direction and the π-π stacking (010) diffraction peaks in the out-of-plane direction with increasing annealing temperature as illustrated in Fig. 5h, i. This drastic improvement in the crystallinity, however, leads to unbalanced charge transport and increased non-geminate recombination, deteriorating performance at higher temperatures, as illustrated in Fig. 5j.

Another important aspect of DCCPs is the linker selection. Alkyl groups connecting the sidechains to the backbones play an important role, and the optimum linker, which is neither small nor the largest, has been shown to enhance the performance of the DCCP [86, 89]. Studies have revealed an inverse relationship between the linker lengths and crystallinity, where shorter linkers led to higher coherence lengths (indicative of greater crystallinity), and longer chains enhanced exciton dissociation. Moderate-sized linkers achieve a good balance between efficient charge separation and manageable crystalline disorder, resulting in optimum performance as illustrated in Fig. 5l. Moreover, charge mobility analysis demonstrates higher and more balanced hole and electron mobilities among all the linkers as shown in Fig. 5k, proving an optimal design.

### Electronic Devices

In the rapidly evolving field of organic electronics, researchers continuously explore novel materials and fabrication techniques to enhance their performance and sustainability. This exploration is driven by the urgent need for environmentally friendly and economically viable alternatives to traditional semiconductor materials to create high-performance, sustainable, and multifunctional electronic devices. This comprehensive study focuses on the electronic applications of CPs, specifically examining their integration in transistors and memory devices to highlight their pivotal role in emerging technologies. Recent studies have highlighted significant advances in various electronic devices, including organic field-effect transistors (OFETs), organic electrochemical transistors (OECTs), charge-trapping memories (CTMs), and resistive random-access memories (ReRAMs or RRAMs). These advancements focus on semiconducting properties and innovative copolymer and device designs.

OFETs have seen improvements by developing new semiconducting polymers that offer higher charge carrier mobility, stability, and processability. These enhancements are crucial for applications requiring flexible displays and sensors. Similarly, OECTs have benefited from advances in polymer electrolytes that improve ion transport efficiency, making them suitable for bioelectronics applications due to their ability to operate efficiently at low voltages. For nonvolatile memory (NVM) devices, CTMs utilize CPs with tailored energy levels to achieve efficient charge storage capabilities. This design innovation enhances data retention times while maintaining low power consumption. RRAMs leverage the unique switching properties of CPs to provide fast switching speeds and high endurance cycles, offering a promising alternative for future data storage solutions. Overall, these developments emphasize the transformative potential of CPs in advancing organic electronics.

#### OECTs & OFETs

We explore a transformative period in semiconducting CPs research, with numerous studies successfully fabricating OFETs and OECTs that show unprecedented performance improvements. These advances prove the viability of these as active components capable of replacing conventional ones. The tunable properties of these polymers enable the fabrication of flexible and lightweight transistors that can operate at low voltages and/or deliver high performance while addressing sustainability issues, integration into current CMOS technologies, and cost concerns. These breakthroughs open new avenues for integrating these materials into the next-generation transistor technologies.

#### Advances in Conductivity and Stability

**Fig. 6:**
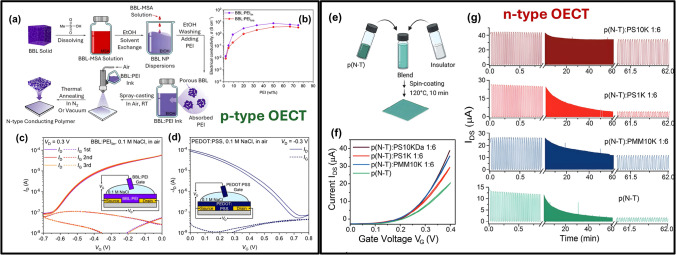
**a** Steps to obtain BBL:PEI ink and deposit it via spray-coating: BBL powder is dissolved in MSA, dispersed in ethanol through solvent-exchange to form BBL nanoparticles, and mixed with PEI to create the final ink. The ink is spray-coated in air and thermally annealed to form a conductive n-type film. **b** Conductivity of BBL:PEIlin and BBL:PEIbra as it varies with PEI content. **c** Transfer curve of a BBL:PEIlin-based OECT in air with 0.1 M NaCl electrolyte shows n-type conduction in depletion-mode and **d** PEDOT:PSS-based OECT shows p-type depletion-mode behavior under similar conditions (**a-d** Reproduced with permission from Ref. [16] Copyright 2021 Springer Nature). **e** Illustration of blending p(N-T):PS/PMM and thin film preparation process. **f** Averaged transfer characteristics for p(N-T), p(N-T):PS 10 K, p(N-T):PS 1 K, and p(N-T):PMM 10 K expressed as monomer ratios; VDS = 0.4 V, average from at least five devices, scan rate ~ 5.36 mV s^−1^. **g** Stability for p(N-T), p(N-T):PS10 K 1:6, p(N-T):PS1 K 1:6, and p(N-T):PMM10 K 1:6; VDS = 0.4 V with ON/OFF cycles at VG steps from 0.4 to 0 V (**e–g** Reproduced with permission from Ref. [62] Copyright 2024 John Wiley and Sons)

This recent progress in semiconducting polymers enabled significant improvements in transistor devices, particularly through enhanced conductivity and overcoming stability issues under operational and environmental stress. Yang et al. (2021) developed a high-conductivity n-type polymeric ink designed for OECTs by blending poly(benzimidazobenzophenanthroline) (BBL) and poly(ethyleneimine) (PEI) (Fig. 6a, b). This ink achieves an impressive electrical conductivity of up to 8 S cm^−1^ and demonstrates robust thermal and ambient stability, maintaining its properties even after 24 h at 200 °C [16]. The BBL:PEI ink can facilitate the creation of high-performance PEDOT OECTs, with applications extending to advanced ternary logic gates due to the device’s unique n-type depletion mode of operation, enhancing its power efficiency with a maximum transconductance of 0.38 mS at zero gate voltage and fast response times (τ_on_ = 167 ms, τ_off_ = 11 ms) extracted from the transfer characteristics shown in Fig. 6c. Other applications include high-performance organic thermoelectric generators (TEGs), demonstrating a record power output of 56 nW per p-n pair at a 50 K temperature gradient.

Although the development of organic polymer blends such as PEDOT:PSS (Fig. 6d) and BBL:PEI (Fig. 6c) enabled conducting materials with major implications for various technologies, including OECTs, the realization of n-type OECTs having comparable performance to p-type transistors remains challenging. Zeglio et al. (2024) showed an enhanced OECT by blending semiconducting n-type polymers with insulating commodity polymers like polystyrene (Fig. 6e) [62]. The goal was to enhance the performance of OECTs by improving the packing of n-type CP chains and reducing the amount of CP needed without sacrificing performance. By mixing p(N-T), an n-type polymer, with high molecular weight polystyrene, they dramatically increased electronic mobility from 0.059 to 1.3 cm^2^ V^−1^ s^−1^ (Fig. 6f).

For a balance between ionic and electronic conductivity, the study also employed different blends of semiconducting n-type polymers, p(C-T), p(C-2 T), and f-BTI2 g-TVTCN, with insulating commodity, polystyrene (PS), with a molecular weight of 10 kDa at a 1:6 monomer ratio. This approach aimed to increase the threshold voltage and improve the mixed ionic/electronic conductivity while retaining the initial drain-source current after over one hour of operation (Fig. 6g). This strategic blending could significantly enhance efficiency and longevity while reducing material costs by minimizing the conjugated polymer required.

**Fig. 7:**
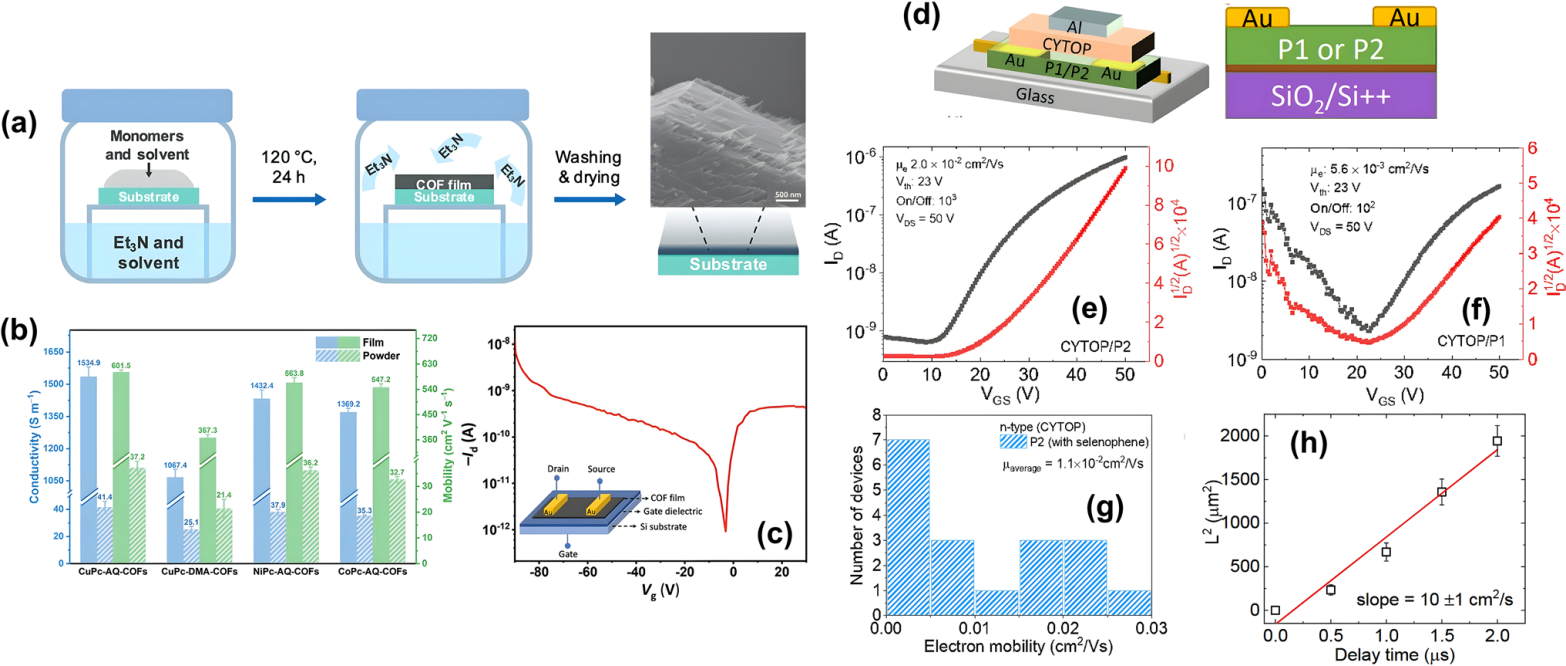
**a** Schematic of the chemical synthesis of CuPc-AQ-COFs and FE-SEM image of CuPc-AQ-COFs film. **b** Conductivity and mobility values of COFs with redox linkers, nonredox linkers, and Ni and Co metal centers: CuPc-AQ-COFs, CuPc-DMA-COFs, NiPc-AQ-COFs, and CoPc-AQ-COFs in both film and powder forms. Error bars mean $\pm$ s.d. (*n* = 5). **c** Transfer plot of the FET device (V_ds_ = −20 V) and schematic illustration of FET devices and Drain and source electrodes under microscopy (**a–c** Reproduced with permission from Ref. [60] Copyright 2024 John Wiley and Sons). **d** Schematic of the top and bottom gate FET. Transfer plot of the top-gate **e** P1 and **f** P2 FET devices. **g** Histogram of electron mobilities from top-gate P2 FETs. **h** The position of the carrier front as a function of the delay (transit) time (**d–h** Reproduced with permission from Ref. [17] Copyright 2022 The Royal Society and Chemistry)

An OFET with exceptional performance was developed by Wang et al. (2024), incorporating a covalent organic framework (COF) using copper-coordinated-fluorinated-phthalocyanine and tetrahydroxy-anthraquinone-based COF films (Fig. 7a) [60]. These COF films were grown through a vapor-assisted synthesis method, exhibiting impressive crystallinity, electrical conductivity of 1.53 × 10^3^ S m^−1^, and hall mobility of 6.02 × 10^2^ cm^2^ V^−1^ s^−1^ at 298 K due to tight interlayer packing that minimized conduction barriers facilitating efficient charge transport (Fig. 7b). OFETs fabricated using these COF films demonstrate high on–off current ratios (> 10^4^), low threshold voltages, and exceptional thermal and chemical stability as depicted in Fig. 7c. The device showed bipolar response to gate modulation with p-type behavior exhibiting higher current than n-type due to the higher hole mobility. Similar recent innovations were also explored by Burmeister et al. (2021) [6] in metal-free two-dimensional covalent organic materials as promising alternatives to traditional semiconductor materials like graphene and silicon for OFETs, emphasizing their environmental and economic benefits by reducing reliance on scarce or toxic metals. These materials allow for tunable electric properties and composition of active components while providing mechanical strength, flexibility, enhanced mobility, recyclability, and environmental and economic benefits by reducing reliance on scarce or toxic metals.

Barron et al. (2022) synthesized thiazole-flanked fluorinated isoindigo (IID) copolymers with thiophene and selenophene substituents to improve charge transport properties and incorporated them in various OFET geometries (Fig. 7d) [17]. This novel design enhanced coplanarity and crystallinity, leading to superior charge transport characteristics. This substitution significantly improved electron mobilities up to 0.25 cm^2^ V^−1^ in top-gate FET architectures with selenophene.s (Fig. 7e-g). They also investigated how replacing sulfur with selenium affects charge transport properties in the FET device using nonlinear optical imaging techniques to visualize charge transport. Electric-field-induced second-harmonic generation (EFISHG) (Fig. 7h) allowed for visualization of carrier mobility and dynamics within the transistor channel, offering insights free from the typical contact resistance issues encountered in traditional electrical measurements.

**Fig. 8:**
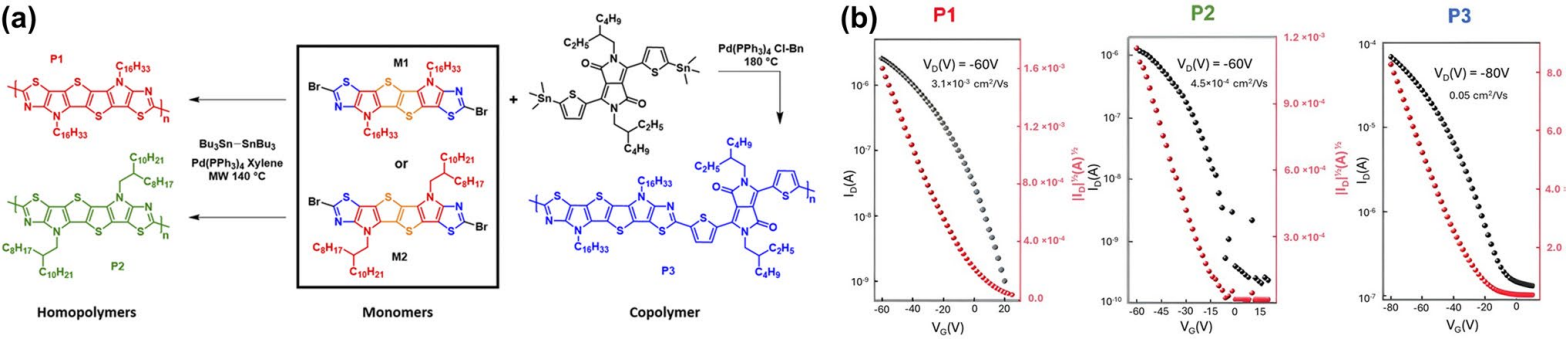
**a** Synthesis of homopolymers P1 and P2 and copolymer P3. **b** Transfer characteristics of the best OTFT devices based on polymers P1, P2, and P3 under optimal conditions (**a** and** b** Reproduced with permission from Ref. [64] Copyright 2022 The Royal Society of Chemistry)

A study by Attar et al. (2022) focused on the synthesizing ladder-type thiazole-fused S,N-heteroacenes as polymeric semiconductors for OFETs, highlighting well-defined fused building blocks based on thiazole moieties (Fig. 8a) [64]. The researchers developed extended conjugation ladder-type heteroacenes with six and nine fused aromatic rings polymerized into homopolymers (P1 and P2) from specific acceptor–donor building blocks. Further copolymerization with diketopyrrolopyrrole units led to the creation of a stepladder copolymer (P3). These polymers demonstrated excellent thermal stability and narrow optical band gaps. The strategic synthesis and design of these polymers improved charge carrier mobility in the fabricated OTFTs, with one showing device mobilities up to 0.05 cm^2^ V^−1^ s^−1^, while the stepladder copolymer exhibited superior performance due to enhanced π-π interactions and packing, achieving the highest mobility among the materials tested when annealed at 200 °C (Fig. 8b). Other synthesized and characterized CPs [90] support this thesis by demonstrating the importance of synthesizing and deposition methods in achieving the desired electrical properties. The study reported electron mobilities as high as 44.7 cm^2^ V^−1^ s^−1^.

#### Structural and Processing Innovations

The development of high-performance transistors using conjugated semiconducting polymers has also seen structural and processing innovations, driven by advances in molecular design, processing techniques, and engineering of CPs for precise control of material structure, improving solubility for solution processing while attaining desired electrical and mechanical properties.

**Fig. 9:**
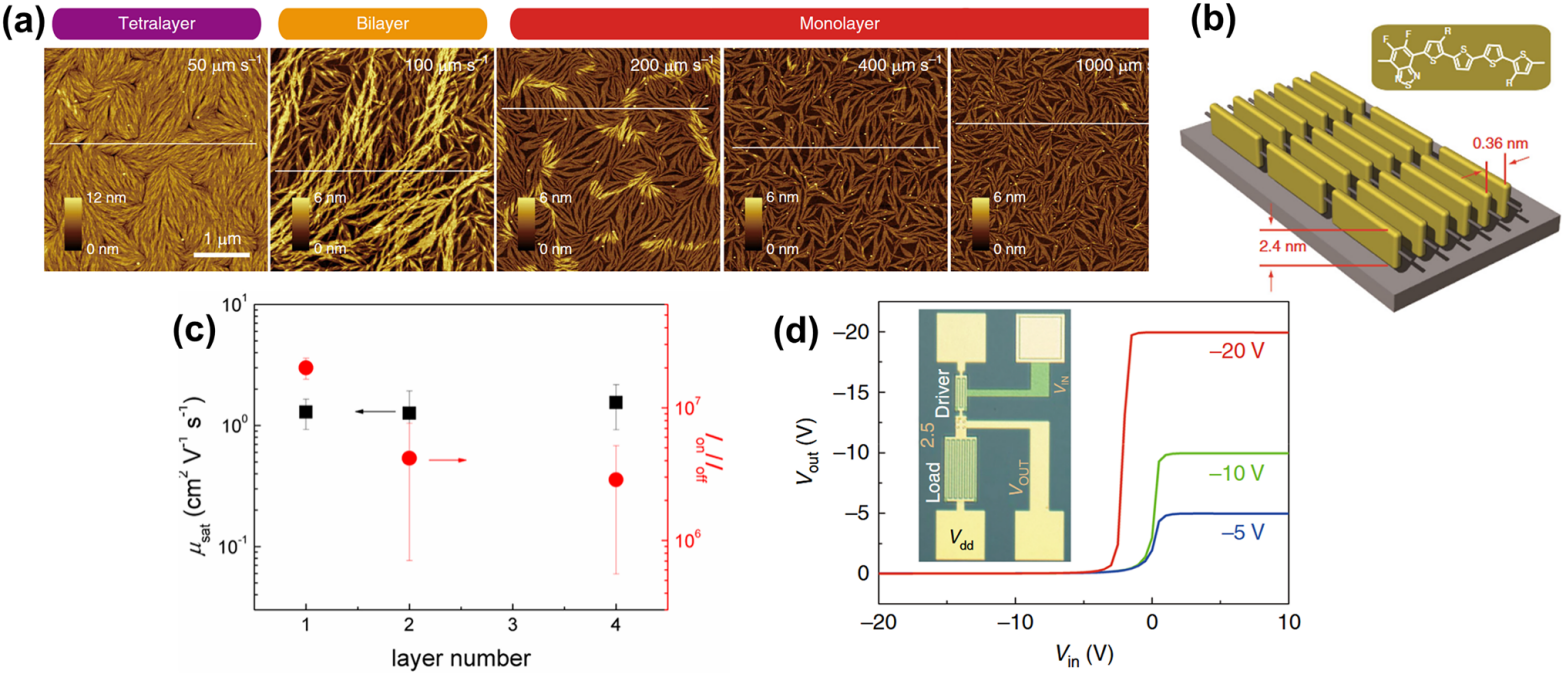
**a** PffBT4 T-2DT ultrathin films from multilayers to monolayers were obtained at dip-coating speeds of 50, 100, 200, 400, and 1000 µm s^−1^ from a 0.5 mg mL^−1^ chloroform solution. **b** Schematic of a PffBT4 T-2DT monolayer nanofiber with edge-on orientation; brown bricks represent PffBT4 T-2DT monomer units. The backbone arrangement is idealized (**a** and **b** Reproduced with permission from Ref. [91] Copyright 2018 Springer Nature). **c** Saturation mobility and on/off ratio versus layer number for PffBT4 T-2DT OFET show that charge carrier transport is layer-independent. No molecular order difference between films of varying thickness is observed. Identical transistor performance in monolayers and multilayers suggests that the first monolayer near the dielectric mainly facilitates charge carrier transport. **d** Integrated circuits based on PffBT4 T-2DT PoM-FETs: Static input–output characteristics of a unipolar inverter with *V*_GS_ = 0 V and *V*_dd_ at −5, −10, and −20 V; inverter layout is shown as an inset (**d** Reproduced with permission from Ref. [31] Copyright 2021 Elsevier)

Understanding molecular structures, solution-state aggregation, and polymer crystal structures is essential for optimizing the thin film’s structure and charge transport properties. Li et al. (2018) [91] successfully fabricated a PffBT4 T-2DT conjugated polymer monolayer OFET using a controlled dip-coating technique on Si substrates with thermally grown SiO_2_ (Fig. 9a). This method achieved high reproducibility and performance due to strong inter-polymer chain interactions that promote edge-on packing and well-defined microstructures. The process yielded high molecular weight polymers with well-ordered nano-fibrillar microstructures, as confirmed by Grazing incident wide-angle X-ray scattering (GIWAXS), which revealed a high degree of molecular ordering in the monolayer with a $\pi$-stacking distance of 0.36 nm (Fig. 9b). The resulting OFETs demonstrated impressive charge carrier mobilities, reaching up to 3 cm^2^ V^−1^ s^−1^ by allowing efficient charge transport due to the precise molecular assembly and dense polymer packing (Fig. 9c), alongside a threshold voltage of 6.5 V and an on/off current ratio of 10^7^. These devices were integrated into unipolar logic gates (Fig. 9d), a seven-stage ring oscillator, and a 15-bit code generator, underscoring their potential for bottom-up organic transistors. In a follow-up study by Li et al. (2021) [31], noncovalent semiconducting polymer monolayers developed using additional solution processing techniques such as Langmuir- Schäfer and spin coating to achieve well-defined crystalline monolayer via π-π stacking interactions. These high-performance OFETs demonstrated improved device mobility. The spin-coated poly(3-alkylthiophene) derivative monolayers achieved a mobility of 1.7 × 10^–2^ cm^2^ V^−1^ s^−1^ at a 3–4 nm thickness, while n-type polymer monolayers films using Langmuir-Schäfer with 3.1 nm thickness reached up to 2.2 × 10^–2^ cm^2^ V^−1^ s^−1^ mobility as the techniques allowed accurate control of long-range order.

**Fig. 10:**
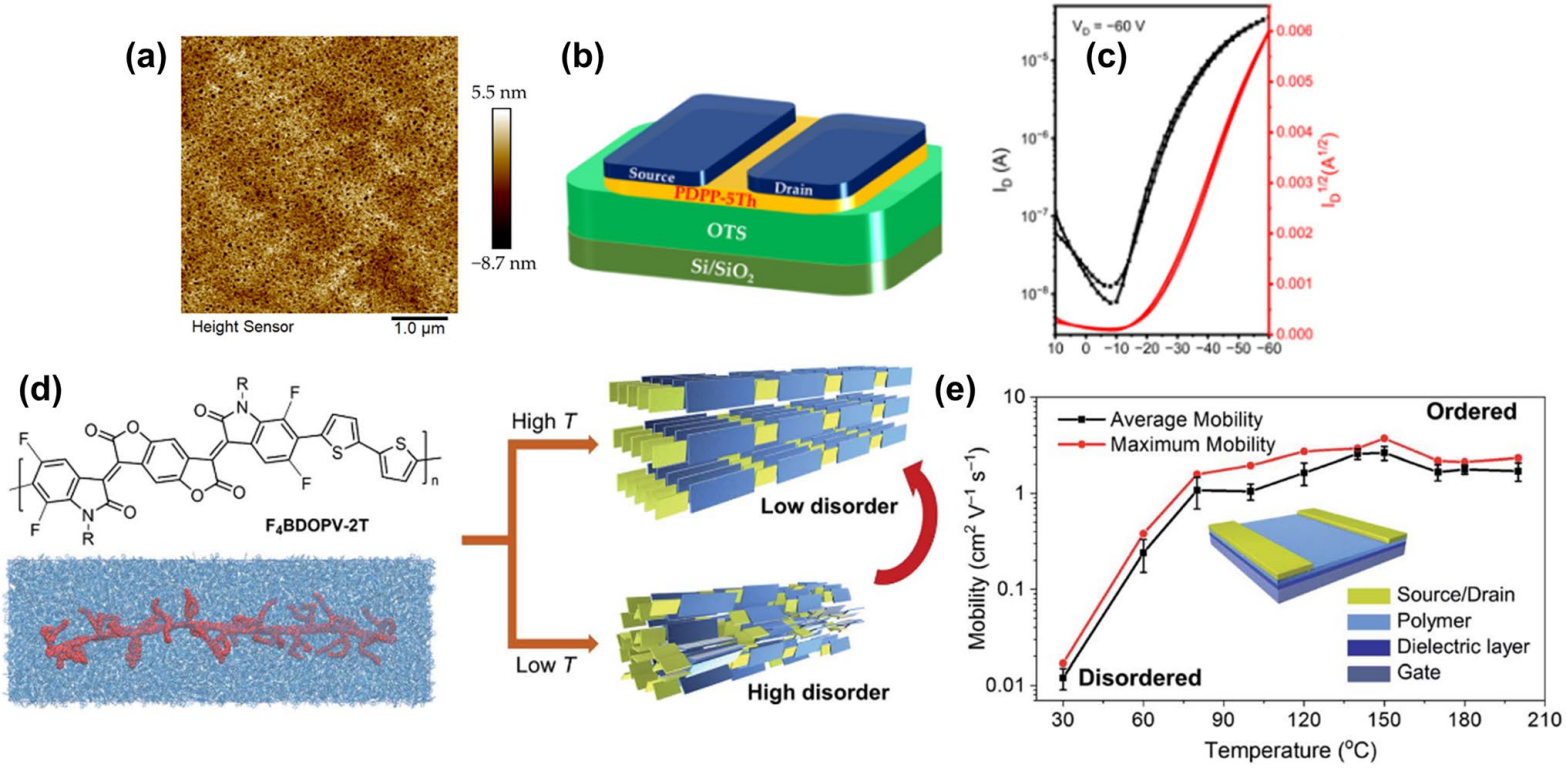
**a** The height images of PDPP-5 Th film from chlorobenzene solution annealed at 180 °C. **b** Ren et al.’s OFET architecture; **c** transport characteristics of Ren et al.’s OFET [92]. **d** Illustration of the ordered microstructures from the polymer solution using temperature-controlled aggregation. **e** Transistor mobility, evaluated from the different polymer thin films (**d** and **e** Reproduced with permission from Ref. [93] Copyright 2020 John Wiley and Sons)

Ren et al. (2024) [92] addressed the challenge of poor solubility in polythiophene systems by synthesizing a novel π-conjugated polymer, PDPP-5 Th, which incorporates diketopyrrolopyrrole (DPP) units with long alkyl side chains into a polythiophene system. This design enhanced solubility and facilitated solution processing for device preparation, improving hole transport properties with mobility of 0.44 cm^2^ V^−1^ s^−1^ due to enhanced donor–acceptor interactions and high crystallinity. The synthesis involved a Stille coupling reaction under palladium catalysis, resulting in a polymer with excellent solubility in chlorinated solvents and high thermal stability up to 380 °C. Density functional theory (DFT) calculations confirmed the coplanarity and stability of the polymer’s structure, promoting efficient carrier migration through strong intramolecular noncovalent interactions. GIWAXS results indicated high crystallinity and edge-on packing mode of the polymer, with a T-Tt stacking distance of 3.63 Å and a *d-d* stacking distance of 20.26 Å, supporting efficient charge carrier hopping between molecular chains. OFET devices fabricated with PDPP-5 Th exhibited excellent p-type transport properties with an on/off current ratio (ION/OFF) of 10^4^, optimized by annealing at different temperatures with the best results at 180 °C (Fig. 10b, c). AFM analysis showed a fibrous intercalation network in the annealed films contributing to the high carrier mobility (Fig. 10a). Yao et al. (2021) provided a comprehensive review of the impact of multi-level microstructures on charge transport in polymer FETs [5], emphasizing the importance of controlling hierarchical microstructures to optimize thin films’ structure and charge transport properties. Their work highlighted that meticulously designed polymers, particularly those that recently employed novel molecular design strategies, have immense potential in leveraging the unique properties of CPs. By focusing on controlling multi-level microstructures from molecular scales to larger formations, they demonstrated that well-engineered molecular and supramolecular structures significantly enhance device functionality. A key innovation was the use of donor–acceptor (DA) polymers, such as F4BDOPV-2 T, which combine electron-donating and electron-accepting units to enhance intermolecular interactions and facilitate efficient charge transport, resulting in air-stable electron mobilities ranging from 1 to 14 cm^2^ V^−1^ s^−1^ [93, 94] (Fig. 10d, e).

**Fig. 11:**
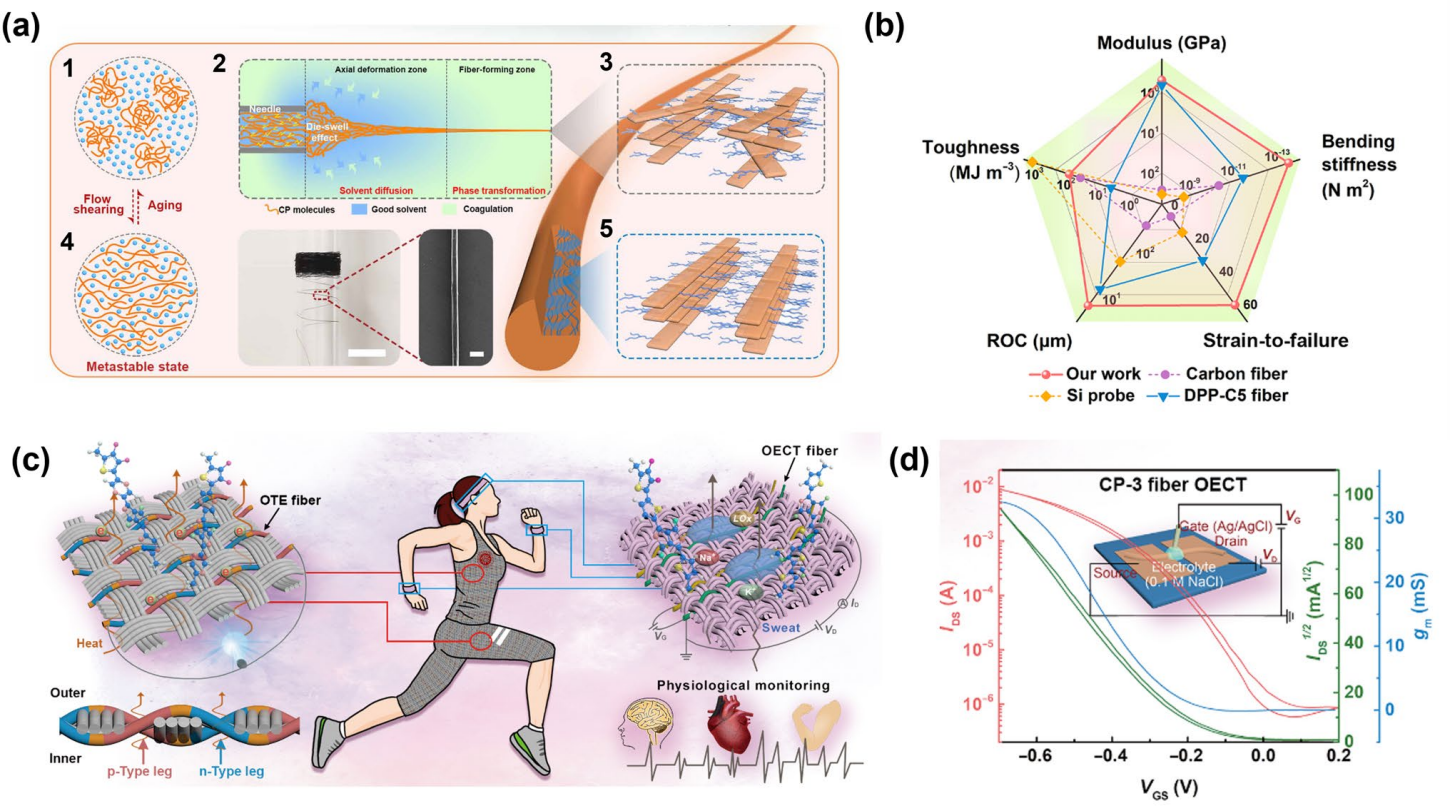
**a** Schematic illustration of the flow-enhanced crystallization (FLEX) mechanism, detailing: (1) original aggregated polymers inside the syringe, (2) solution flow disaggregating and aligning semiconducting polymers in the needle, (3) slow solvent diffusion and exchange in the coagulation bath enhancing crystallinity, and stages (4) before and (5) after drawing/stretching. Insets include a photograph and SEM image of fibers obtained through continuous production. **b** Comparison of key mechanical properties among various fibrous materials: carbon fiber, Si probe, dPP-c5 fiber, and our fiber. **c** Schematic diagram illustrating potential wearable applications of semiconducting polymer fibers. **d** Representative p-type transfer characteristic and corresponding gm curves for a fiber OECT with W/L = 15 µm/20 µm [95]

Zhang et al.’s (2024) study introduces the flow-enhanced crystallization (FLEX) method [95] for continuous production of high-performance semiconducting polymer fibers, addressing the challenge of fabricating CP fibers with excellent mechanical and electronic properties (Fig. 11). Using the polymer solution in low concentration and utilizing strong extensional and shear flows, the researchers achieved polymer disaggregation and alignment, resulting in fibers with remarkable tensile strength exceeding 200 MPa and toughness surpassing 80 MJ m^−3^. These mechanical properties are an order of magnitude better than traditional semiconducting polymer fibers and films, rivaling many synthetic fibers. The FLEX-produced fibers exhibit exceptional performance as stretchable conductors, with Herman’s orientation factor (ƒ) increasing from 0.42 to 0.74 as needle diameter decreases or length increases, highlighting the improved chain alignment and crystallinity.

The FLEX method improves mechanical properties and leads to unique strain-enhanced electronic properties. The study shows that fibers produced using FLEX have low density (0.96 g cm^−3^) comparable to polypropylene and an effective bending stiffness ranging from 0.7 to 1.3 nN m, similar to neural tissues. This makes them ideal for implantable high-performance bioelectronics. This was demonstrated in OECTs based on FLEX-fibers, which exhibited high transconductance (1.2 mS) and low threshold voltage (0.2 V). The fibers’ ability to maintain conductivity under stretching conditions further highlights the strain-enhanced electronic performance, with conductivity remaining stable at strains up to 50%. When stretched from 0 to 50%, ƒ increased from 0.67 to 0.91, indicating further enhanced crystallinity. The method’s ability to maintain and enhance electronic properties while dramatically improving mechanical characteristics opens up new possibilities for integrating functional fibers into wearable and implantable electronics. This work represents a significant advancement over previous attempts to improve CP fiber properties, such as melt-processing approaches for DPP-C5 polymers, which resulted in moderate tensile strength [96] and compromised electronic performance.

#### Novel Materials and Mechanisms

**Fig. 12:**
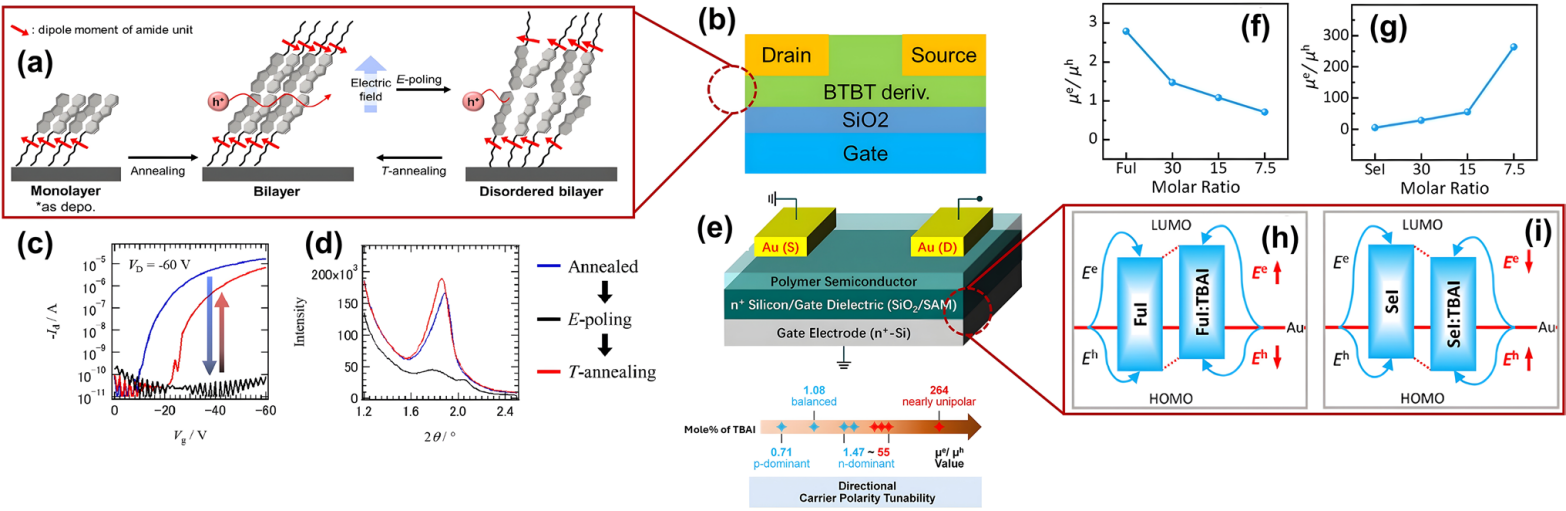
**a** Monolayer periodicity at 303 K, bilayer periodicity after annealing at 443 K, and disordered bilayer structure post E-poling with *V*_G_ = −70 V at 443 K. The electric field was perpendicular to the substrate surface, while the macro dipole moment of the 1D N–H·O = hydrogen bonding chain was not parallel to it. **b** Sambe et al. OFET device schematic. **c** Transfer curves of the initial device (blue), E-poling (black), and T-annealing (red). **d** OP-XRD pattern changes in a sandwich-type device: initial (blue), E-poling (black), and T-annealing (red) (**a, c** and **d** Reproduced with permission from Ref. [31] Copyright 2024 American Chemical Society). **e** Liu et al. OFET device schematic. Trends in mobility values (µ_h_ and µ_e_) and µ_e_/µ_h_ ratios for OFETs using **f** FuI:TBAI and **g** SeI:TBAI films, with a schematic of charge carrier injection barrier changes for **h** FuI and **i** SeI polymers (**e-i** Reproduced with permission from Ref. [99] Copyright 2022 American Chemical Society)

The ability to fine-tune the electronic properties of the polymers on demand leads to devices that can be specifically optimized for various electronic applications, making them highly versatile compared to current devices. Liu et al. (2023) introduced a novel mechanism that allows for the tunability of carrier polarity in ambipolar OFET, illustrated in Fig. 12e, using polymers based on diketopyrrolopyrrole (DPP) and bithiophene imide (BTI), with furan (FuI) and selenophene (SeI) as flanking group [97]. By utilizing side-chain engineering, they were able to control the balance between hole and electron transport, which is revolutionary for developing high-performance ambipolar OFETs. The devices made from FuI (Fig. 12f, h) and SeI (Fig. 12g, i) exhibit significant differences in performance based on the molecular modifications and the presence of the ionic additive. The introduction of tetrabutylammonium iodide (TBAI) as an ionic additive further enhanced this capability, transforming furan-based devices from n-type dominant to balanced charge transport. This tunability is essential for the design and functionality of OFETs for logic and complementary circuits.

Similarly, integrating molecular dynamics within organic semiconductors can achieve multifunctional properties for developing stimuli-responsive organic electronic materials adaptive to varying external conditions. Sambe et al. (2024) demonstrated controlled switching of carrier transport through ferroelectric polarization manipulation [23]. They exploited the ferroelectric properties of a novel 2D alkylamide-substituted benzothieno[3,2-b]benzothiophene (BTBT) derivative (BTBT-NHCOC14H29), which opens up possibilities for designing organic electronic devices with switchable carrier transport features. Upon thermal annealing at 443 K, the BTBT derivative transitioned from a monolayer to a bilayer structure, enhancing its p-type semiconducting channel hole mobility to 10^–3^ cm^2^ V^−1^ s^−1^. This integration achieves multifunctional properties suitable for advanced electronics applications such as memory or sensing technologies; Sambe’s team demonstrated an OFET device (Fig. 12b) with reversible switching capabilities under electric field poling or thermal annealing conditions as depicted in Fig. 12a, c, d.

These advancements in next-generation CP materials are capable of revolutionizing transistors capabilities across various applications such as wearable technologies, logic circuits, smart textiles, and chemical sensors among others. However, advancing our understanding of semiconducting polymers’ role in modern electronics by addressing key challenges related to conductivity, stability, cost-effectiveness, and integration still requires future innovations in CP’s flexibility, manufacturing sustainability, and integration with current CMOS processes. By continuing to explore innovative approaches alongside meticulous molecular design, we can contribute toward more sustainable developments across various domains within the modern-day electronics industry while addressing critical challenges traditionally faced thereby ensuring high-performance outcomes aligned closely alongside ecological and cost considerations. The highlights of the work explored in the review for CPs used in OECT and OFET are given in Table 3.

**Table 3:** Summary of performance metrics

Polymer	Type	Mobility (cm^2^ V^−1^ s^−1^)	Conductivity (S cm^−1^)	Ref/Year
PffBT4 T-2DT	Device	3.02 (hole)	–	[91]/2018
F4BDOPV-2 T	Device	14 (electron)	–	[5]/2020
BBL/PEI	Film	–	8	[6]/2021
Polymer bar coated nanofiber	Device	3.02	–	[31]/2021
Ni3(HTB)2	Film	–	160	[54]/2021
Cu3(HTB)	Film	–	2500	[54]/2021
Fe3(HTTP)2	Film	4200	–	[54]/2021
FUI	Device	0.0092 (electron)	–	[97]/2022
SEI	Device	0.154 (electron)	–	[97]/2022
CuPc-AQ-COF	Film	602	1530	[60]/2023
BTBT-NHCOC14H29	Device	0.001 (hole)	–	[23]/2024
BBL	Device	–	2.744 S	[98]/2024
s-SWNTs/n-CPs	Device	0.5 (hole)	–	[15]/2024

#### Nonvolatile Memory

Nonvolatile memory (NVM) devices are essential components in modern electronics, enabling data retention without power and driving advancements in data storage, neuromorphic computing, and sustainable memory systems. CPs, with their versatile and tunable properties, have been harnessed in NVM technologies to enhance key performance metrics such as ON/OFF current ratios, retention times, switching voltages, and endurance cycles. Recent developments have concentrated on leveraging CPs in both charge-trapping memories (CTMs) and resistive random-access memories (ReRAMs), while addressing challenges related to scalability, stability, and technological integration.

CPs offer distinct advantages due to their capacity to support discrete charge-trapping sites through molecular design, facilitating efficient charge storage and release. The delocalized $\pi$-electron systems intrinsic to CPs enable the formation of shallow and deep trap states within the polymer matrix. In CTMs, devices typically employ a metal insulator semiconductor (MIS) structure, utilizing an insulating CP layer to trap charges under an applied electric field. Innovative material designs have allowed the CP layer to function either as the charge-trapping medium or the tunnel/blocking layers. By engineering the molecular structure of CPs, researchers can optimize trap density and distribution, thereby improving memory characteristics. Advances such as incorporating multiple dielectric interlayers and utilizing top-gate configurations have further enhanced the performance, stability, and scalability of these memory devices.

ReRAMs, characterized by their simple metal–insulator–metal (MIM) structure and potential for high-density integration, represent another promising class of NVM devices. In CP-based ReRAMs, resistive switching behavior is facilitated by redox-active sites within the polymer. The application of external voltage induces oxidation and reduction processes, enabling reversible switching between high-resistance states (HRS) and low-resistance states (LRS) for binary data storage. Incorporating redox-active units such as viologens, ferrocene, or quinones into CP backbones imparts the ability to undergo reversible redox reactions, which is crucial for reliable switching performance. To enhance ReRAM devices stability and repeatability, strategies such as cross-linking CP chains or doping with substances like iodine or metal ions have been employed to introduce additional charge transport pathways. These modifications result in more stable and consistent switching cycles. Furthermore, blending CPs with insulating polymers or incorporating inorganic nanoparticles refines control over filament formation and distribution, enhancing both scalability and performance consistency. Utilizing techniques such as self-assembled monolayers (SAMs) allows precise control over the thickness and morphology of the switching layers. Electrode configurations, including top–bottom and lateral arrangements, can also be optimized to improve switching characteristics, with the choice of electrode material playing a critical role in charge injection and filament formation.

Integrating CPs into NVM devices like CTMs and ReRAMs presents significant opportunities to enhance memory performance through molecular design and device engineering. Ongoing research into material innovations and device architecture is essential to overcome scalability, stability, and integration challenges for next-generation high-performance, flexible, and sustainable memory technologies.

#### Two-Dimensional CPs

Due to their unique planar structures and superior charge transport properties, two-dimensional conjugated polymers (2D CPs) have attracted considerable attention for their potential in CTMs and ReRAMs. Their ultrathin nature and tunable electronic characteristics render them ideal candidates for applications such as neuromorphic computing. Recent advancements have focused on eco-friendly synthesis methods and incorporating functional groups to enhance device performance and environmental compatibility.

**Fig. 13:**
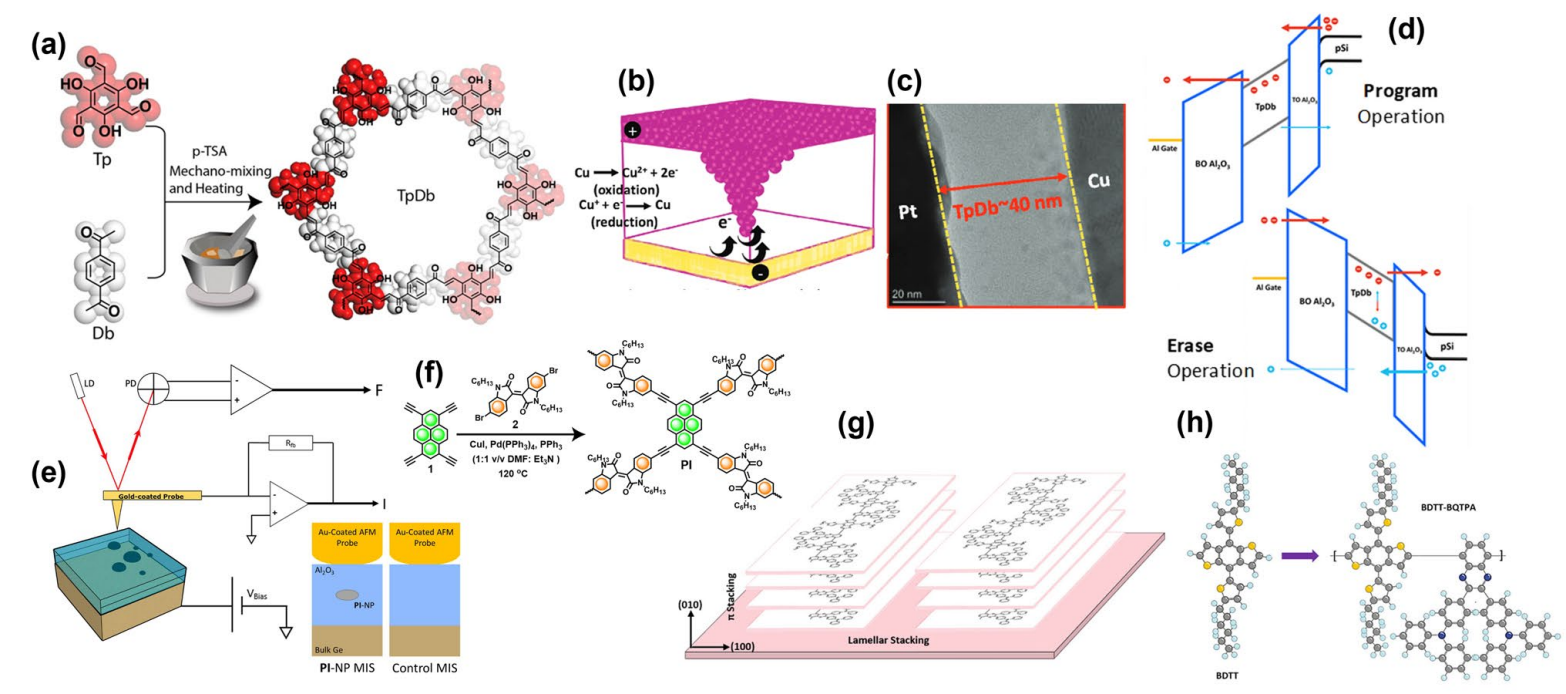
**a** TbDb synthesis scheme [101]. **b** Electrochemical processes involved in resistive switching; **c** Cross-section TEM images of the sandwiched TpDb (**b** and **c** Reproduced with permission from Ref. [100] Copyright 2024 John Wiley and Sons). **d** Energy band diagram of a MOS capacitor during CTM program/erase operations [100]. **e** Circuit for surface mapping and electrical probing of a single 2D CP using cAFM, with inset showing MIS stack cross section; **f** Synthetic approach for Pyrene-Isoindigo 2D-CMP (**e** and **f** Reproduced with permission from Ref. [101] Copyright 2023 Springer Nature). **g** Molecular structures of 2D conjugated BDTT electron donor and the BDTT-BQTPA unit. **h** Schematic of π-π stacking in PBDTT-BQTPA polymer chains (**g** and **h** Reproduced with permission from Ref. [102] Copyright 2021 Springer Nature)

Pal et al. [99] investigated carbonyl-decorated two-dimensional polymers (TpDb) for ReRAM devices (Fig. 13a–c), emphasizing their potential for sustainability and biocompatibility. The TpDb polymer is synthesized via a green aldol condensation, forming a conjugated enone framework with a redox-active triol core. These TpDb polymers feature ultramicro channels with carbonyl (C = O) and hydroxyl (O–H) groups that promote charge localization through interlayer hydrogen bonding. This design supports robust performance even at elevated temperatures resulting in excellent bipolar resistive switching behavior. Such properties mimic synaptic plasticity, making TpDb-based devices suitable for neuromorphic computing tasks like image denoising and edge detection. The devices achieved a large resistive switching window of 10^3^, low programming/erasing voltages, and exhibited high endurance and retention. Using potentially fully recyclable electrodes underscores their environmental impact, addressing e-waste concerns. Additionally, these devices significantly lower operational voltages, thereby reducing power consumption and positioning them as efficient components for sustainable artificial intelligence applications.

Al-Ajeil et al. [100] employed the same TpDb as a novel charge-trapping layer for MIS capacitive memory devices (Fig. 13d). Continuing the emphasis on sustainability, these polymers, constructed from coupled *sp*^2^ carbon atoms, offer a low-toxicity alternative to traditional inorganic materials. TpDb-based devices exhibit a substantial memory window of 3.2 V and operate at low programming/erasing voltages, demonstrating excellent endurance (104 cycles) and stable retention over 10^4^ s at 100 °C. The inherent memory hysteresis of TpDb significantly contributes to its durability and retention, affirming its promise as a candidate for NVM applications. Moreover, TpDb shows over a 52% increase in memory window compared to previously reported devices while maintaining lower operational voltages. Its charge-trapping efficiency is likely enhanced by interlayer hydrogen bonding and structural defects.

Extending the application of 2D CPs to the nanoscale, Rezk et al. [101] demonstrated a nanoscale charge-trapping device by integrating pyrene and indigo-based 2D conjugated microporous polymer (2D-CMP) nanoparticles into a MIS structure, probed using conductive atomic force microscopy (cAFM) (Fig. 13e, f). The device achieved a wide memory window of 1.5 V at low operational voltages (~ 1 V), with retention times exceeding 10^4^ s and endurance of 10^3^ cycles. The 2D-CMPs act as nano-floating gates, optimizing charge confinement and enabling high-density, low-power memory applications. The superior scalability and flexibility of these devices are particularly advantageous for developing deformable, low-power NVM devices.

Progressing toward high-yield and uniform thin-film devices, Zhang et al. [102] introduced 2D CPs in ReRAM devices, enhancing π-π stacking and crystallinity with thiophene derivatives, leading to improved coplanarity and uniformity in thin films (Fig. 13g, f). The polymer PBDTT-BQTPA exhibits nonvolatile bipolar resistive switching behavior with uniformity across the film, resulting in a high production yield of about 90% and minimal device variation. These devices demonstrate exceptional scalability and robustness, maintaining performance at nanometer scales with excellent retention (over 10^4^ s) and endurance (exceeding 10^8^ cycles), even under high temperatures. Low operational voltages (~ ± 0.30 V) make them suitable for neuromorphic computing applications, including Boolean logic operations and binary neural network construction.

#### Advances CP Architectures

The evolution of advanced CP architecture has opened new avenues for enhancing the performance and functionality of many memory devices. By tailoring the molecular structures and incorporating functional groups or unique building blocks, researchers have achieved improved charge-trapping mechanisms, multi-level storage capabilities, and enhanced environmental stability. These innovations contribute to realizing high-density, low-power, and durable memory devices suitable for various applications.

**Fig. 14:**
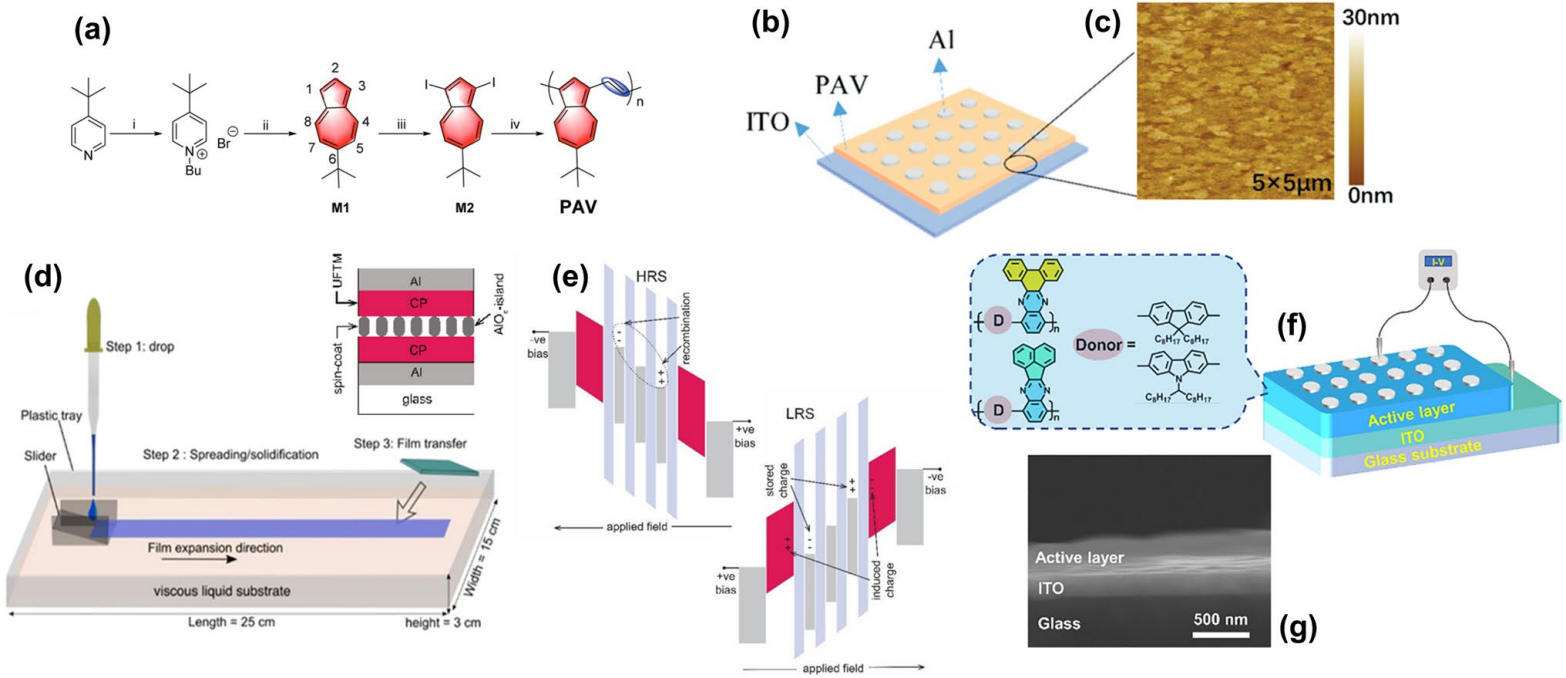
**a** Synthesis of the azulene-based polymer PAV; **b** diagram of the Al/PAV/ITO device; **c** AFM image of the PAV film on ITO (**a-c** Reproduced with permission from Ref. [103] Copyright 2022 The Royal Society of Chemistry). **d** UFTM fabrication steps with an inset of the device architecture and their. **e** Energy diagram under applied bias (d and e Reproduced with permission from Ref. [104] Copyright 2023 Elsevier). **f** Schematic of quinoxaline-based sandwich structure and **g** SEM cross section of the device (**f** and **g** Reproduced with permission from Ref. [105] Copyright 2023 Elsevier)

Azulene-based CPs (PAV), known for forming stable azulenium cation radicals that facilitate reversible charge trapping, have shown promise in memory devices with hole-dominated conduction [103]. In situ, UV–Vis, and electron paramagnetic resonance spectroscopy confirmed the efficiency of radical cation dynamics within the azulene structure under an electric field. The Al/PAV/ITO device demonstrates stable switching voltages of ± 1.8 V, attributed to these dynamics. The devices also exhibit high endurance over 500 cycles with long-term retention exceeding 104 s (Fig. 14a–c). The narrow voltage distributions indicate the efficiency of radical cation formation as a charge-trapping mechanism. Despite an ON/OFF ratio of 102, improvements in environmental stability are desired compared to devices employing covalent functionalization. The polymer’s sensitivity to pH changes and electric fields suggests potential new functionalities.

Utilizing innovative fabrication techniques, Sharma et al. [104] employed the unidirectional floating-film transfer method (UFTM) to fabricate layer-by-layer thin films of CPs such as PTB-7, PBTTT, and RR-P3HT (Fig. 14d, e). UFTM allows multilayer coatings with controlled conformation without damaging underlying layers, ensuring a face-on orientation in PTB-7 films, which optimizes vertical charge transport. The PTB-7 devices achieved impressive ON/OFF ratios of 106 and current densities up to 10 mA, attributed to high crystallinity. Additionally, PTB-7 exhibits bistable resistive switching behavior. The UFTM process maintains film integrity and alignment, leading to enhanced device durability and performance without the need for complex material synthesis.

In further demonstrations of multi-level memory systems, He et al. [105] developed nonvolatile ternary memory devices using quinoxaline-based CPs incorporating phenanthrene and acenaphthene moieties as charge-trapping sites (Fig. 14f, g). These polymers enable ternary memory systems due to their unique charge transport mechanisms and multi-level storage capabilities, driven by intramolecular charge transfer (ICT). The study highlights the role of quinoxaline and phenanthrene/acenaphthene as electron-withdrawing centers that create distinct resistive states (OFF, ON1, ON2) through ICT, enabling multi-level memory effects. The ReRAM device exhibits enhanced data storage density with ternary switching behavior, supported by high ON/OFF ratios (> 10^4^) and low operational voltages (0.64 V). They demonstrate excellent reproducibility and stability over 10^3^ cycles and retention times of 10^4^ s. Density functional theory (DFT) simulations confirm these findings, showing electronic structures favorable for ICT and effective charge trapping through HOMO localization in fluorene/carbazole groups and LUMO association with quinoxaline/acenaphthene groups.

#### Metal Coordination and Composite CPs

Incorporating metal coordination and composite structures into CPs has proven a highly effective strategy for enhancing memory device performance. Researchers have developed devices exhibiting improved stability, charge-trapping efficiency, and environmental resilience by integrating the unique properties of metals, nanoparticles, or other functional materials with CPs. These composite materials facilitate the design of memory devices that meet the demands of modern NVM devices, including low power consumption, high endurance, and scalability.

**Fig. 15:**
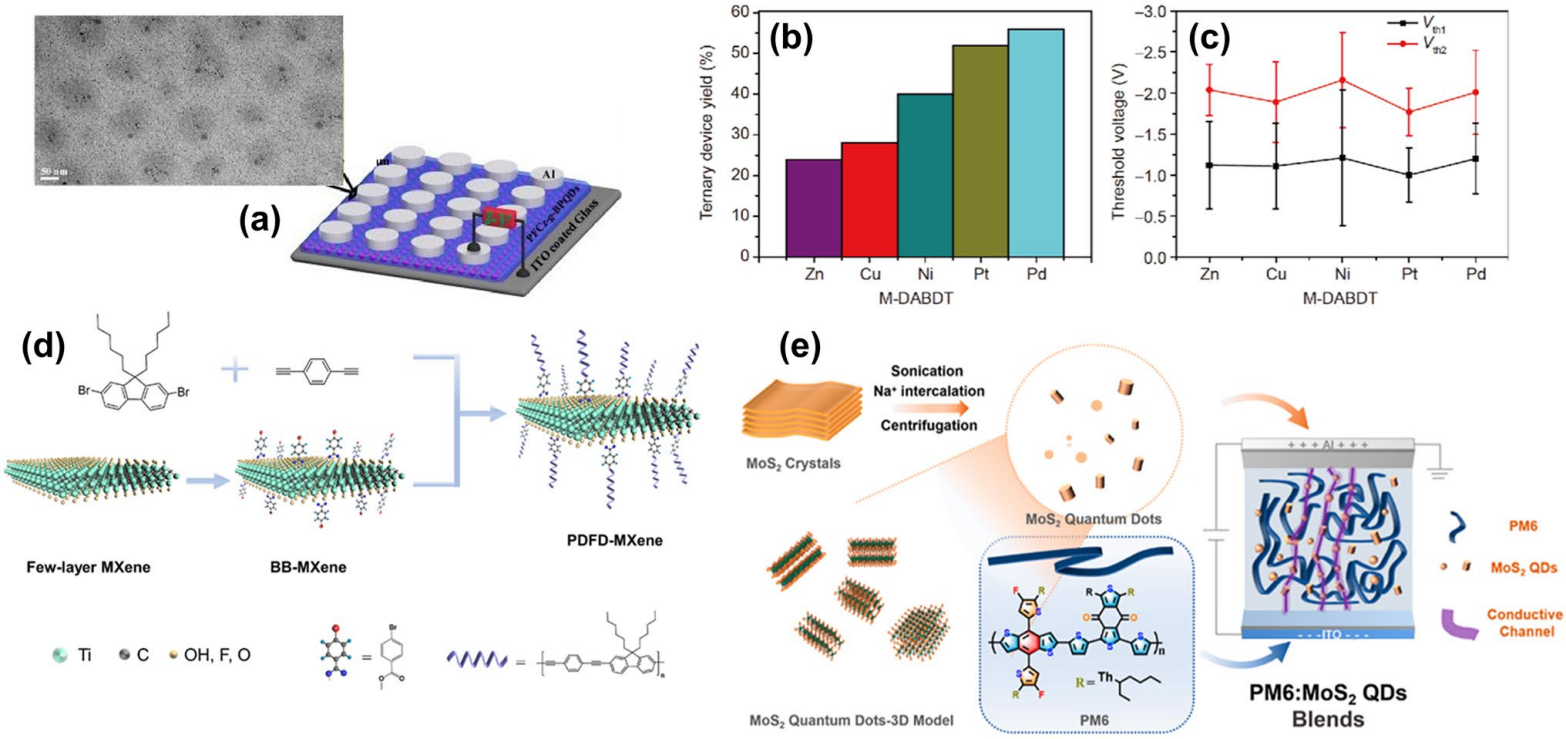
**a** HRTEM image of PFCz-g-BPQDs alongside the structure of the Al/active material/ITO memory device (**a** Reproduced with permission from Ref. [106] Copyright 2019 The Royal Society of Chemistry). **b** Ternary device yield and **c** average threshold voltages (Vth_1_ and Vth_2_) for M-DABDT-based memory devices from a batch of 50 devices (**b** and **c** Reproduced with permission from Ref. [107] Copyright 2019 Springer Nature). **d** Synthesis process of PDFD-Mxene (**d** Reproduced with permission from Ref. [108] Copyright 2023 American Chemical Society). **e** Preparation of MoS_2_ QDs, their integration into a blended system with PM6 in CHCl_3_ solution, and the device resistance mechanism highlighting MoS_2_ QDs as nodes controlling conductive channel formation and rupture (**e** Reproduced with permission from Ref. [109] Copyright 2023 American Chemical Society)

Cao et al. [106] introduced a novel method to enhance memory device performance by covalently functionalizing black phosphorus quantum dots (BPQDs) with the CP poly(9,9-dioctylfluorene-co-benzothiadiazole) (PFCz) (Fig. 15a). This approach improves environmental stability and mitigates BPQD degradation under oxygen and moisture through a robust P–C bond formed via a one-step synthesis. The resulting RRAM devices exhibit an exceptional ON/OFF current ratio over 10^7^ across 600 cycles at low operational voltages, ideal for low-power applications. Covalent functionalization enhances charge-trapping efficiency and longevity, making these devices suitable for extended durability applications.

Expanding on metal coordination, Cheng et al. [107] synthesized one-dimensional π-*d* conjugated coordination polymers (1D CCPs) for ReRAM devices using various metal ions (Zn^2+^, Cu^2+^, Ni^2+^, Pt^2+^, Pd^2+^) with a 2,5-diaminobenzene-1,4-dithiol (DABDT) linker (Fig. 15b, c). These materials achieved low threshold voltages, and ternary device yields up to 56%. The enhanced performance is attributed to structural planarity and improved crystallinity of 1D CCPs, optimizing charge transport through space-charge-limited current (SCLC) and grain-boundary-limited current (GBLC) mechanisms. The smaller hole injection barriers facilitate hole-dominated charge transport. Planar structures with smaller band gaps enhance performance by reducing defect-based losses, with Pd-DABDT structures showing optimal results.

The integration of CPs with 2D materials is also explored by Sun et al. [108], who investigated the incorporation of poly[(9,9-dihexyl-9H-fluorene)-alt-(1,4-diethynylbenzene)] (PDFD) with MXene (Ti_3_C_2_T_x_) (Fig. 15d). The resulting composites demonstrate that the covalent attachment of PDFD to MXene improves stability and solubility, leading to an impressive ON/OFF ratio exceeding 10^4^. This functionalization reduces the operational voltage and enhances device longevity. The PDFD-MXene composite maintains a stable resistive switching window under 50% humidity for 60 days due to robust covalent bonds that prevent degradation. Annealing at 150 °C in N_2_ further optimizes performance by enhancing crystallization and charge transfer efficiency. The switching mechanism is driven by an electric field-induced charge transfer process, ensuring a stable and reversible conductive state.

Further demonstrating the benefits of integrating CPs with nanocomposites, Zhang et al. [109] showed that the incorporation of molybdenum disulfide quantum dots (MoS_2_ QDs) into PM6 polymers enhances the stability and performance of ReRAM devices (Fig. 15e). The QDs serve as electron-trapping centers, forming stable conductive channels and improving the reliability of switching behavior. These PM6-MoS_2_ QD-based devices enable multistage conductance states and synaptic functions such as long-term potentiation (LTP) and short-term depression (STD), which are crucial for neuromorphic computing. The devices achieved an ON/OFF ratio of 30 with narrow switching voltage ranges (0.6/−2.4 V), indicating improved stability and scalability. Additionally, including MoS_2_ QDs enhances cycling longevity and ensures environmental and thermal stability, making these devices suitable for real-world applications.

The advancements in NVM devices utilizing CPs have significantly improved performance metrics such as ON/OFF ratios, retention times, switching voltages, and environmental stability. Through strategic material design and engineering, challenges related to scalability, stability, and device longevity are being addressed. The development of CTMs and ReRAMs underscores the potential of CPs to revolutionize data storage technologies, paving the way for next-generation memory devices with enhanced functionality and sustainability. Moreover, the highlights of the work explored in the review for CPs used in CTM and ReRAM are presented in Table 4.

**Table 4:** Summary of performance metrics for various NVM devices

Material System	Type	Memory Window (V)	Operational Voltage (V)	ON/OFF Ratio	Endurance (Cycles)	Retention	Ref/Year
AL/PAV/ITO	ReRAM	∼3.6	+ 1.80/–1.85	102	> 500	> 104	[103]/2022
PFCz-g-BPQDs	ReRAM	∼2.84	−0.89/+ 1.95	> 107	> 600	> 104	[106]/2019
2D-CMP in MIS	CTM	1–1.5	< 1	105	> 103	> 104	[101]/2023
TpDb in MIS	CTM	3.2	± 2/± 3	–	> 104	> 104	[100]/2024
Cu/TpDb/Pt	ReRAM	3.5	−1.5/2	103	> 104	> 104	[99]/2024
PDFD-MXene	ReRAM	–	–	104	60 days	–	[108]/2023
Quinoxaline	ReRAM	–	0.64	103	–	104	[105]/2023
PBDTT-BQTPA	ReRAM	–	± 0.30	103	> 108	> 104	[102]/2021

## Outlook

Conjugated polymers (CPs) are strategically positioned to transform modern electronics and photonics, fueled by continuous progress in molecular design, synthesis methodologies, and device integration. Their solution processability and inherent flexibility are key to revolutionizing organic electronics, especially in devices like OFETs, OECTs, and CP-based NVM. Recent molecular engineering breakthroughs have yielded CPs with enhanced charge carrier mobilities and improved environmental stability. This enables the development of high-performance devices with low-voltage operation and neuromorphic computing potential, mimicking synaptic functions essential for artificial neural networks. These advancements pave the way for applications in flexible displays, wearable electronics, bioelectronics, high-density data storage, and even medical applications like phototherapies and targeted drug delivery systems leveraging reactive oxygen species generation upon light exposure. In OPVs, donor–acceptor CPs have achieved certified PCEs up to 19% in laboratory-scale devices. Chlorinated non-fullerene acceptors further enhance performance by improving intramolecular charge transfer and broadening light absorption into the near-infrared region. Research into SCOSCs with non-fullerene acceptors offers paths to even higher efficiencies, although upscaling active areas while maintaining performance remains a challenge.

Despite these significant scientific advancements, realizing widespread commercialization requires addressing critical economic and technical hurdles. Scalability and cost remain paramount concerns; the intricate complicated synthesis and purification of high-performance CPs compared to silicon hinders mass adoption [45]. Long-term environmental stability is crucial, especially for OPVs facing weatherability issues and degradation from moisture, oxygen, and UV exposure. Multilayer encapsulation using materials like atomic-layer-deposited Al_2_O_3_ and UV-curable resins is extending operational lifetimes to around 7,000 h (equivalent to 5 years of outdoor use) [110], but further improvements are needed. The field must effectively bridge the gap between high-performance materials demonstrated in laboratories and commercially viable devices by optimizing device architectures, establishing scalable, low-cost manufacturing processes like slot-die coating, blade-coating, and roll-to-roll processing, and strategically targeting niche applications where CPs’ unique attributes, such as flexibility, tunability, and biocompatibility, offer distinct advantages.

Despite these significant scientific advancements, realizing widespread commercialization requires addressing critical economic and technical hurdles. Scalability and cost remain paramount concerns; the intricate complicated synthesis and purification of high-performance CPs compared to silicon hinders mass adoption [45]. Long-term environmental stability is crucial, especially for OPVs facing weatherability issues and degradation from moisture, oxygen, and UV exposure. Multilayer encapsulation using materials like atomic-layer-deposited Al_2_O_3_ and UV-curable resins is extending operational lifetimes to around 7,000 h (equivalent to 5 years of outdoor use) [110], but further improvements are needed. The field must effectively bridge the gap between high-performance materials demonstrated in laboratories and commercially viable devices by optimizing device architectures, establishing scalable, low-cost manufacturing processes like slot-die coating, blade-coating, and roll-to-roll processing, and strategically targeting niche applications where CPs’ unique attributes, such as flexibility, tunability, and biocompatibility, offer distinct advantages.

Future research should prioritize developing CPs with enhanced environmental stability without sacrificing performance, exploring cost-effective, large-scale fabrication techniques including bio-inspired and green chemistry approaches, and investigating hybrid systems that combine the benefits of CPs with the robustness of inorganic materials. Developing stretchable and self-healing properties in CPs could also unlock durable flexible devices for wearables [111]. The global market for CPs, currently valued at $3.6 billion in 2023 and projected to reach $7.1 billion by 2030 with a compound annual growth rate of 9.3% [112], is propelled by escalating demands in flexible electronics, sustainable energy solutions like building-integrated photovoltaics (BIPV), and high-performance memory and sensor devices, particularly in health care and consumer electronics. While OPVs still lag behind perovskite and silicon single-junction solar cells in efficiency, OFET-based memory devices and flexible sensors for wearable health monitoring patches are closer to near-term commercialization, potentially capturing niche markets and a 5%–7% share of the $58 billion nonvolatile memory market by 2030 [112]. However, realizing the expansive potential of CPs necessitates the development of unified manufacturing standards to ensure device reproducibility and reliability across different production lines. Overcoming technical barriers, reducing costs to below $50 kg^−1^ for bulk CP synthesis, and navigating evolving regulatory and environmental considerations, including the focus on bio-based content and halogenated solvent restrictions, will be crucial. Moreover, the ultimate impact of CPs hinges on successfully translating molecular-level innovations into scalable, cost-competitive products, demanding a concerted effort of scientific ingenuity, industrial collaboration, and strategic market positioning to carve out and expand niches where their unique properties offer unparalleled value and drive the next wave of technological advancements.

## Declarations

### Use of Large Language Models

The authors used OpenAI’s GPT-4o to proofread the vocabulary and grammar, ensuring clearer language. They also reviewed the model’s output to confirm that it retained technical and scientific accuracy.
